# Elliptical *β* ‐barrel deformation underlies gating in VDAC1

**DOI:** 10.1002/pro.70677

**Published:** 2026-06-19

**Authors:** L. Bergdoll, M. Elgeti, J. Belyaeva, A. Zlobin, J. P. Duneau, W. Hubbell, J. Abramson

**Affiliations:** ^1^ Laboratoire d'Ingénierie des Systèmes Macromoléculaires CNRS, UMR 7255—Aix Marseille Université Marseille France; ^2^ Institute for Drug Discovery and Institute for Medical Physics and Biophysics, Leipzig University, Faculty of Medicine Leipzig Germany; ^3^ Institute for Drug Discovery, Leipzig University, Faculty of Medicine Leipzig Germany; ^4^ Stein Eye Institute, David Geffen School of Medicine University of California, Los Angeles Los Angeles California USA; ^5^ Department of Physiology, David Geffen School of Medicine University of California, Los Angeles Los Angeles California USA

**Keywords:** DEER, gating, MD simulations, pressure perturbation, VDAC

## Abstract

Gating by voltage‐dependent anion channels (VDAC) regulates mitochondrial metabolite flux, yet the structural mechanism underlying the open‐to‐closed transition remains unresolved. Here, we combine atomistic molecular dynamics (MD) simulations with double electron–electron resonance (DEER), using hydrostatic pressure as a reversible thermodynamic perturbation to shift conformational equilibria and stabilize low‐population states. MD simulations reveal localized intrinsic flexibility within *β*‐strands *β*1–*β*5 and *β*19, as well as in cytosolic loops connecting *β*6–*β*7 and *β*8–*β*9. High‐pressure DEER measurements in lipid nanodiscs corroborate these predictions, identifying reversible, pressure‐dependent distance changes within the pore lumen consistent with asymmetric deformation of the *β*‐barrel. DEER‐informed analysis of unbiased MD trajectories reveals an elliptical *β*‐barrel conformation aligned parallel to the N‐terminal helix that corresponds to the pressure‐stabilized experimental state. ATP permeation simulations identify a free‐energy barrier to metabolite translocation in this elliptical geometry, whereas diffusion through the circular open state is energetically favorable. These findings indicate that the elliptical conformation represents a transient gating‐competent state rather than a fully closed channel. Together, our results support a gating mechanism driven by reversible *β*‐barrel deformation and establish pressure‐perturbed DEER integrated with MD as a general strategy for capturing transient, functionally relevant conformations of membrane channels.

## 1 INTRODUCTION

Embedded in the mitochondrial outer membrane (MOM), the voltage‐dependent anion channel (VDAC) serves as the primary conduit for ion and metabolite exchange between the cytosol and mitochondria, facilitating the transport of ATP, ADP, pyruvate, and other metabolites essential for oxidative phosphorylation and cellular bioenergetics (Bergdoll et al., 2017; Rostovtseva & Colombini, 1997). VDAC is a dynamic channel, transitioning between a well‐defined single open state and multiple distinct closed states, exerting profound control over mitochondrial activity (Lemasters & Holmuhamedov, 2006; Queralt‐Martin et al., 2019). In the open state, VDAC supports efficient metabolite flux across the MOM, whereas closure sharply restricts metabolite transport and limits ATP and ADP exchange (Rostovtseva & Colombini, 1996). Through these transitions, VDAC directly regulates mitochondrial energy output. Despite the importance of this process, the structural mechanism underlying VDAC gating remains unresolved. Understanding this mechanism is particularly important given the involvement of VDAC dysfunction in cancers (Heslop et al., 2021; Yuan et al., 2025), neurodegenerative diseases (Verma et al., 2022), and metabolic disorders (Varughese et al., 2021).

High‐resolution structures of VDAC in the open conformation revealed a 19‐strand *β*‐barrel architecture containing an N‐terminal *α*‐helix positioned within the pore lumen (Bayrhuber et al., 2008; Hiller et al., 2008; Ujwal et al., 2008). In contrast, the structure of the closed state has yet to be determined, but early models proposed that voltage gating involves large‐scale displacement or partial unfolding of the N‐terminal segment. However, electrophysiological measurements and crosslinking studies have predominantly disfavored this mechanism, instead implicating structural plasticity of the *β*‐barrel itself, including elliptical deformation, as a contributor to gating (Teijido et al., 2012; Zachariae et al., 2012). Consistent with this view, recent molecular‐dynamics simulations indicate localized *β*‐strand flexibility rather than wholesales displacement of the N‐terminal helix (Ngo et al., 2022). While large N‐terminal ejection from the pore is unlikely, smaller rearrangements, such as helix rotation or subtle barrel deformation, remain possible and may occur either independently or in concert. Discriminating between N‐terminal rearrangement and *β*‐barrel deformation therefore remains a central challenge, particularly because the closed state is transient, sparsely populated, and typically accessed only under perturbative conditions such as applied voltage, reduced pH, mechanical stress, or interaction with partner proteins (Colombini, 2004;  Rostovtseva et al., 2006; Teijido et al., 2014).

Here, we combine molecular‐dynamics simulations with double electron–electron resonance (DEER) to probe conformational changes associated with VDAC1 gating. DEER measures nanometer‐scale distances between site‐directed spin labels and is well suited for resolving conformational ensembles of membrane proteins in lipidic or micellar environments. To stabilize low‐population states relevant to gating, we applied hydrostatic pressure, a perturbation known to shift structural equilibria toward conformations with reduced partial molar volume (Akasaka, 2003; Lerch et al., 2014, 2020). This approach allows direct experimental access to otherwise elusive VDAC conformations and enables quantitative evaluation. Comparison with MD simulations provides atomistic insight into rare conformations of VDAC which may contribute to gating. Using this combined strategy, we find that VDAC1 retains a globally stable *β*‐barrel architecture, in the absence of destabilizing mutations, while undergoing reversible, localized conformational changes that produce ellipsoidal barrel geometries. These deformations restrict the pore and are consistent with functional closed‐state behavior (i.e., reduced ion conductance and obstructed ATP flux), providing experimental support for *β*‐barrel deformation as a central element of VDAC gating.

## 2 RESULTS

### 2.1 MD simulations identify localized flexibility in the open VDAC1 *β*‐barrel

To characterize the conformational landscape accessible to VDAC1 in its open state, we performed all‐atom molecular dynamics (MD) simulations to identify regions exhibiting elevated intrinsic flexibility. VDAC1 adopts a 19‐stranded *β*‐barrel architecture with an N‐terminal *α*‐helical segment positioned within the pore and oriented parallel to the membrane surface within the pore lumen (Bayrhuber et al., 2008; Hiller et al., 2008; Ujwal et al., 2008). Notably, VDAC1 is the first membrane‐integrated *β*‐barrel protein identified with an odd number of strands, an architecture proposed to confer enhanced conformational plasticity and support gating transitions (Teijido et al., 2012; Zeth & Thein, 2010). We therefore simulated VDAC1 embedded in a lipid bilayer representative of the mitochondrial outer membrane, with the goal of sampling conformational heterogeneity and identifying structural elements most likely to participate in gating‐associated rearrangements.

**FIGURE 1:**
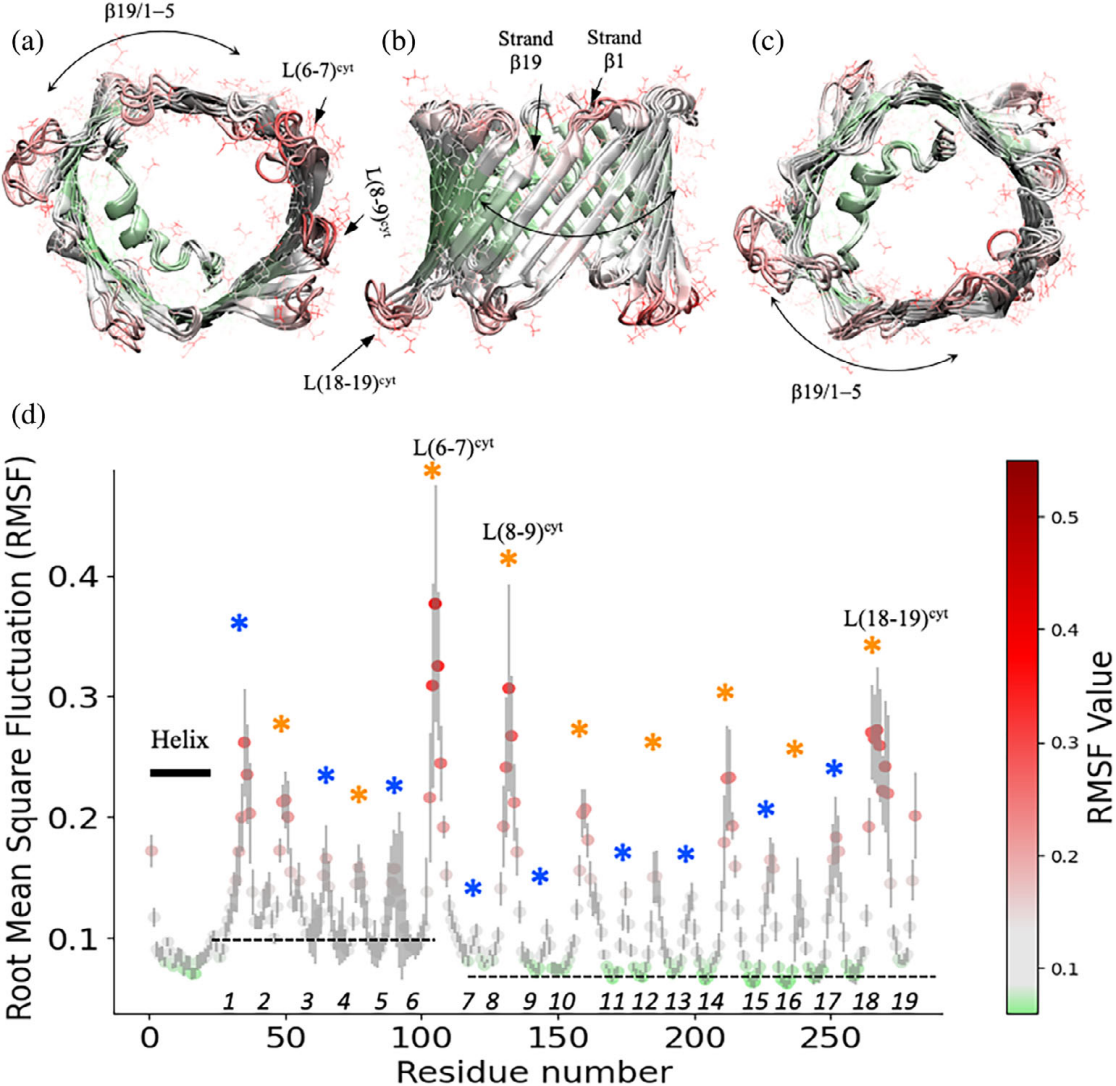
Localized flexibility of VDAC1 revealed by all‐atom molecular dynamics simulations. Five representative structures of VDAC1 sampled over a 500‐ns all‐atom molecular dynamics simulation are shown and color‐coded according to backbone root‐mean‐square fluctuations (RMSF). Structures are viewed from (a) the cytoplasmic side, (b) the *β*19–*β*1–*β*5 face, and (c) the intermembrane space (IMS) side. Elevated RMSF values are localized to one side of the *β*‐barrel, encompassing *β*‐strands *β*1–*β*5 and the structurally contiguous *β*19 strand, as well as select cytosolic loop regions. (d) RMSF values for all residues (backbone atoms only) plotted as a function of residue number. Cytosolic loops are indicated by orange asterisks, and IMS‐facing loops by blue asterisks. Numbers on top of the *x*‐axis represent the *β*‐strands. Regions corresponding to the N‐terminal helix exhibit comparatively low RMSF values, consistent with a globally stable *β*‐barrel scaffold and localized conformational flexibility.

Figure 1 displays backbone root‐mean‐square fluctuations (RMSF) calculated over a 500‐ns simulation, which report the average positional deviation of each residue and provide a measure of local flexibility. While the N‐terminal helix and *β*‐strands *β*6–*β*18 remained comparatively rigid, elevated RMSF values were observed on one side of the barrel, encompassing *β*‐strands *β*1–*β*5 and the structurally contiguous *β*19 strand. In addition, pronounced fluctuations were detected in cytosolic loops connecting *β*‐strands 6–7 [L(6–7)^cyt^] and *β*‐strands 8–9 [L(8–9)^cyt^]. Notably, these cytosolic loops displayed greater flexibility than loops facing the intermembrane side of the channel, consistent with their proposed role in accommodating interactions with cytosolic partner proteins (Rosencrans et al., 2025; Rostovtseva & Bezrukov, 2008). These features were reproducible across six independent simulations (Figure 1; SFig. 1), indicating that enhanced flexibility in these regions reflects intrinsic properties of the VDAC1 structure rather than stochastic fluctuations.

This pattern of flexibility closely mirrors experimental observations from crystallographic studies. A recent comparison of B‐factors from cryogenic and room‐temperature crystal structures of VDAC1 revealed the largest temperature‐dependent differences in the intracellular portions of *β*‐strands *β*1, *β*2, *β*5, and *β*6, as well as in cytosolic loop regions L(6–7)^cyt^ and L(8–9)^cyt^ (Gonzalez‐DeWhitt et al., 2024). The concordance between MD‐derived RMSF profiles and experimentally observed B‐factor variations supports a consistent picture in which VDAC1 maintains a globally stable *β*‐barrel scaffold while exhibiting localized flexibility in specific *β*‐strands and cytosolic loops. These regions therefore represent prime candidates for conformational rearrangements associated with VDAC gating and provide a structural framework for the selection and interpretation of site‐directed spin labeling (SDSL) positions used in subsequent DEER experiments.

### 2.2 Structural analysis in lipidic and micellar environments

**FIGURE 2:**
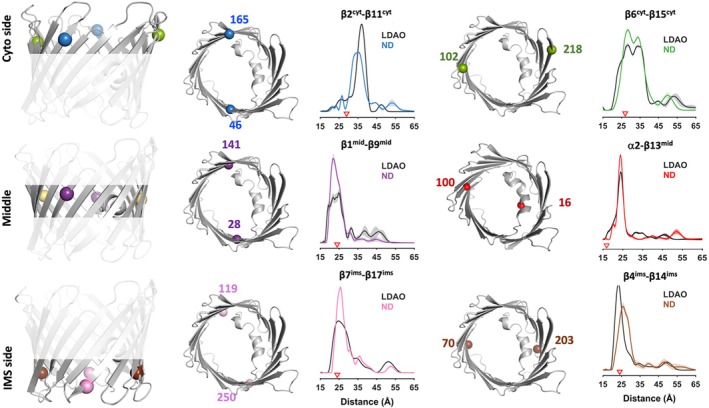
Comparison of VDAC1 structure in lipid nanodiscs and detergent micelles by DEER spectroscopy. SDSL and DEER spectroscopy were used to compare conformational properties of VDAC1 reconstituted in lipid nanodiscs (ND) or solubilized in LDAO micelles. Six double‐cysteine variants were introduced at strategic locations spanning the cytosolic face *β*2^cyt^–*β*11^cyt^, *β*6^cyt^–*β*15^cyt^, the mid‐pore *α*2–*β*13^mid^, *β*1^mid^–*β*9^mid^, and the intermembrane space (ims) side *β*4^ims^–*β*14^ims^, *β*7^ims^–*β*17^ims^. For each pair, the modeled spin‐label positions are shown on the VDAC1 structure (PDB: 3EMN), alongside the corresponding DEER‐derived distance distributions for LDAO (black) and nanodiscs (colored traces). Red triangles indicate MDDS‐predicted distances derived from the crystal structure. Associated dipolar evolution data are shown in SFig. 2.

To quantify conformational flexibility across defined regions of the VDAC1 pore, we employed SDSL and DEER spectroscopy. Six double‐cysteine variants were generated to probe distinct structural regions: the cytosolic surface *β*2^cyt^–*β*11^cyt^ and *β*6^cyt^–*β*15^cyt^, the mid‐pore region *α*2–*β*13^mid^ and *β*1^mid^–*β*9 ^mid^, and the intermembrane space (ims) side with *β*4^ims^–*β*14^ims^ and *β*7^ims^–*β*17^ims^ (Figure 2). Expected DEER distances were calculated from the murine VDAC1 structure (PDB 3EMN) using MD with dummy spin labels (MDDS) (Qi et al., 2020), a computational approach that incorporates spin‐labels in protein structural models and derives distance distributions from short MD simulations. These predictions provide reference values for interpreting experimental DEER data.

To assess how the membrane environment influences VDAC1 structure and dynamics, we compared DEER measurements from proteins reconstituted into lipid nanodiscs with those obtained from samples solubilized in LDAO micelles. While LDAO is widely used for structural studies of VDAC, nanodiscs provide a native‐like bilayer environment and minimize oligomerization artifacts that can complicate DEER analysis. Across all six spin‐label pairs, the dominant DEER peaks closely matched MDDS‐predicted distances in both detergent and lipidic environments (Figure 2 and SFig. 2), indicating that the crystal structure and both experimental conditions represent the open‐state *β*‐barrel.

Despite this global similarity, nanodisc‐reconstituted samples generally exhibited narrower distance distributions than their detergent‐solubilized counterparts, consistent with reduced conformational dynamics. This effect was particularly evident for *β*2^cyt^–*β*11^cyt^ and *β*7^ims^–*β*17^ims^ spin pairs (Figure 2 and SFig. 2), suggesting that lipid bilayer interactions stabilize specific regions of the barrel. In contrast, the pair *β*4^ims^–*β*14^ims^ displayed modest broadening in nanodiscs, indicative of enhanced flexibility at the IMS interface in a bilayer environment.

Together, these results demonstrate that while detergent and nanodisc environments yield highly similar average structures, lipid bilayers modestly dampen conformational heterogeneity and provide a more stable platform for probing subtle, pressure‐induced rearrangements of VDAC1.

### 2.3 Pressure perturbation reveals conformational changes in VDAC1

Under hydrostatic pressure, LDAO‐solubilized samples exhibited reversible oligomerization that interfered with interpretation of single‐channel behavior (SFig. 3). This oligomerization introduces intermolecular DEER contributions that obscure intramolecular distance changes and preclude reliable analysis of conformational states. For this reason, all subsequent pressure‐perturbation experiments were performed exclusively using nanodisc‐reconstituted VDAC1. To probe alternate conformations potentially involved in VDAC1 gating, we subjected nanodisc‐reconstituted samples to 3 kbar hydrostatic pressure. Application of pressure favors protein conformations with reduced partial molar volume by promoting side‐chain repacking and changes in hydration, thereby shifting the structural equilibrium toward low‐population states. Hydrostatic pressure has proven particularly effective for revealing conformational heterogeneity and sparsely populated intermediates in membrane proteins, including pressure‐induced shifts between functional states (Gonzalez‐DeWhitt et al., 2024; Lerch et al., 2020) that are not detectable under ambient conditions, and is used here as a non‐physiological perturbation to probe the conformational landscape rather than to represent a pressure‐driven biological mechanism. Consistent with this, the DEER distance distributions reflect pressure‐induced population shifts within a heterogeneous conformational ensemble, observed as broadening and asymmetric redistribution rather than a uniform shift in average distances.

**FIGURE 3:**
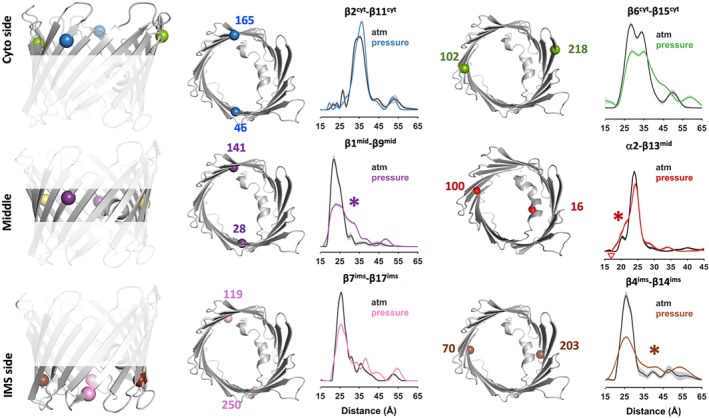
Pressure perturbation reveals local conformational changes in VDAC1. SDSL and DEER spectroscopy were used to monitor pressure‐induced conformational changes in nanodisc‐reconstituted VDAC1 at 3 kbar hydrostatic pressure. Six double‐cysteine mutant pairs were introduced at strategic locations spanning the cytosolic face *β*2^cyt^–*β*11^cyt^, *β*6^cyt^–*β*15^cyt^, the mid‐pore *α*2^mid^–*β*13^mid^, *β*1^mid^–*β*9^mid^, and the intermembrane space (IMS) side *β*4^ims^‐14^ims^, *β*7^ims^–17^ims^. For each spin pair, DEER‐derived distance distributions are shown at atmospheric pressure (black) and under 3 kbar pressure (colored traces). Asterisks (*) indicate spin pairs exhibiting significant but reversible pressure‐induced changes. Associated dipolar evolution data are shown in SFig. 9.

Application of 3 kbar hydrostatic pressure revealed significant distance changes at four of the six positions (Figure 3): *β*6^cyt^–*β*15^cyt^, *β*1^mid^–*β*9^mid^, *α*2–*β*13^mid^, and *β*4^ims^–*β*14^ims^. The remaining spin pairs—*β*2^cyt^–*β*11^cyt^ and *β*7^ims^–*β*17^ims^—were largely unchanged, consistent with a rigid *β*‐barrel scaffold in these regions. Pressure broadened the distance distributions for *β*6^cyt^–*β*15^cyt^ and *β*4^ims^–*β*14^ims^, indicating increased flexibility at both membrane interfaces. Notably, the two mid‐pore pairs shifted in opposite directions, with *β*1^mid^–*β*9^mid^ shifting toward longer distances and *α*2–*β*13^mid^ toward shorter distances. These asymmetric changes are consistent with a pressure‐stabilized deformation of the barrel cross‐section at the mid‐pore. All distance changes were reversible upon pressure release (SFig. 4), confirming that these conformations coexist with the open state under ambient conditions.

The observation that pressure selectively stabilizes asymmetric mid‐pore rearrangements, while leaving other regions unchanged, supports a model in which VDAC gating involves structural adaptation rather than global unfolding. Such pressure‐stabilized asymmetry is consistent with *β*‐barrel deformation mechanisms previously proposed on the basis of electrophysiological measurements (Teijido et al., 2012) and MD simulations (Zachariae et al., 2012).

The N‐terminal domain of VDAC1, comprising the *α*1 and *α*2 segments, resides within the pore and has long been implicated in channel gating through its influence on pore electrostatics and barrel stability (Mertins et al., 2012; Teijido et al., 2012). Recent MD simulations by Ngo et al. demonstrated that charged residues within the *α*2 segment participate in a voltage‐sensitive electrostatic network, and that perturbation of this network induces subtle deformations of the *β*‐barrel sufficient to promote channel closure (Ngo et al., 2022). Importantly, this mechanism does not require extensive movement of the *α*2 segment itself, but instead highlights its role in coupling local electrostatic perturbations to collective barrel rearrangements. Consistent with this model, Najbauer et al. reported destabilization of the *α*2 region accompanied by small *β*‐barrel deformations during gating in a quintuple VDAC mutant (Najbauer et al., 2022). Together, these studies suggest that the *α*2 segment, anchored to the barrel wall through hydrophobic interactions, plays a central role in gating by transducing small local perturbations into subtle, collective deformations of the *β*‐barrel.

In line with this framework, we measured the wall‐to‐helix distance between a stable mid‐pore barrel position and the *α*2 segment *α*2–*β*13^mid^. Under hydrostatic pressure, this distance distribution developed a reproducible ~4 Å shorter shoulder (Figure 3) indicating a pressure‐stabilized rearrangement involving the *α*2 region. While this change could reflect modest helix repositioning or rotation, its magnitude and reversibility are also consistent with local barrel deformation coupled to *α*2 motion. Because changes in DEER distance distributions may reflect both backbone rearrangements and altered spin‐label rotamers, we used MD simulations to gain structural insight into the conformational changes most consistent with the observed pressure‐induced distance shifts.

**FIGURE 4:**
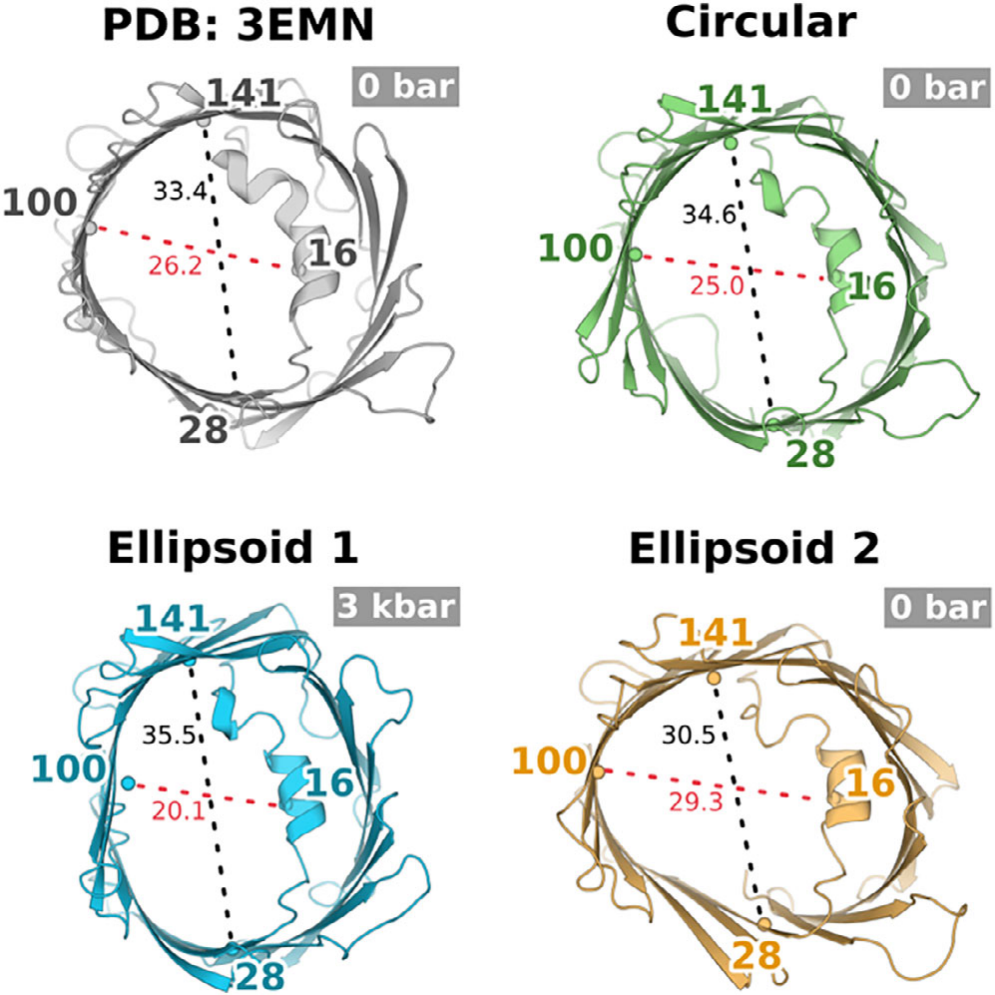
DEER‐consistent MD ensembles reveal elliptical VDAC1 conformations. Average structures representing the near‐circular *β*‐barrel conformation (green) and two elliptical conformations—ellipsoid 1 (blue) and ellipsoid 2 (orange) identified by DEER‐informed filtering of unbiased MD simulations—are shown in comparison with the open‐state crystal structure of VDAC1 (gray; PDB 3EMN). Distances corresponding to experimentally probed mid‐pore DEER spin‐label pairs are indicated, illustrating axis‐specific barrel deformations. The ellipsoid 1 conformation corresponds to the pressure‐stabilized DEER state, whereas the ellipsoid 2 conformation aligns with the ambient‐pressure DEER state.

### 2.4 DEER‐informed ensemble analysis reveals elliptical VDAC1 conformations

To obtain structural insight into the pressure‐dependent changes observed in the DEER experiments, we applied a DEER‐informed analysis to our MD simulations (Belyaeva & Elgeti, 2024). The MD trajectories were run without any applied pressure, voltage, or DEER‐derived restraints and therefore sample the conformational heterogeneity intrinsic to the open‐state energy landscape. Experimental DEER distance distributions were used post hoc to identify subsets of simulation frames most consistent with the experimentally observed pressure‐stabilized or ambient‐pressure states. The DEER‐informed filtering and clustering procedure is summarized in SFig. 5.

For the DEER‐informed analysis of the MD trajectories, we used mid‐pore distance constraints from *β*1^mid^–*β*9^mid^ and *α*2–*β*13^mid^ variants that exhibited reproducible, pressure‐dependent shifts in the experimental distributions. For each MD trajectory, spin labels were attached in silico to the simulated frames, and spin–spin DEER distance distributions were simulated using the chiLife Python package (Tessmer & Stoll, 2023). The simulated DEER distributions from individual frames were then averaged across each complete trajectory to characterize the overall VDAC1 conformation sampled in that trajectory.

Across five independent MD trajectories, three reproduced the experimental trends observed under either pressure or ambient conditions. One trajectory (T2) yielded distance distributions consistent with the pressure‐stabilized DEER state, whereas two trajectories matched the ambient‐pressure DEER state (T1 and T4). The remaining trajectories did not reproduce distance distributions consistent with the experimental DEER data, and were therefore not used to describe pressure‐associated conformational ensembles. This outcome was expected, as the simulations were not biased in any way to reach low‐population, or pressure‐stabilized conformations.

Because DEER distance distributions reflect contributions from both backbone rearrangements and spin‐label rotameric flexibility, we applied a filtering procedure to minimize the influence of local rotamer effects and emphasize global conformational differences. Simulation frames exhibiting DEER‐consistent distances, characteristic of the pressure‐stabilized population, were selected and subsequently clustered (SFig. 5). This analysis ensured that the resulting ensembles reflect coordinated rearrangements of protein segments rather than fluctuations of individual spin labels.

Clustering of the filtered frames revealed three dominant conformational ensembles. The most populated ensemble corresponds to a near‐circular *β*‐barrel geometry closely resembling the crystallographic open state (PDB 3EMN). Two additional ensembles exhibited elliptical barrel geometries, which we term ellipsoid 1 and ellipsoid 2, with elongation axes parallel or orthogonal to the N‐terminal helix, respectively (Figure 4). The ellipsoid 1 ensemble aligned with the pressure‐stabilized DEER distance distributions, whereas the ellipsoid 2 ensemble corresponded to the ambient‐pressure DEER state, consistent with reversible, axis‐specific barrel deformation.

**FIGURE 5:**
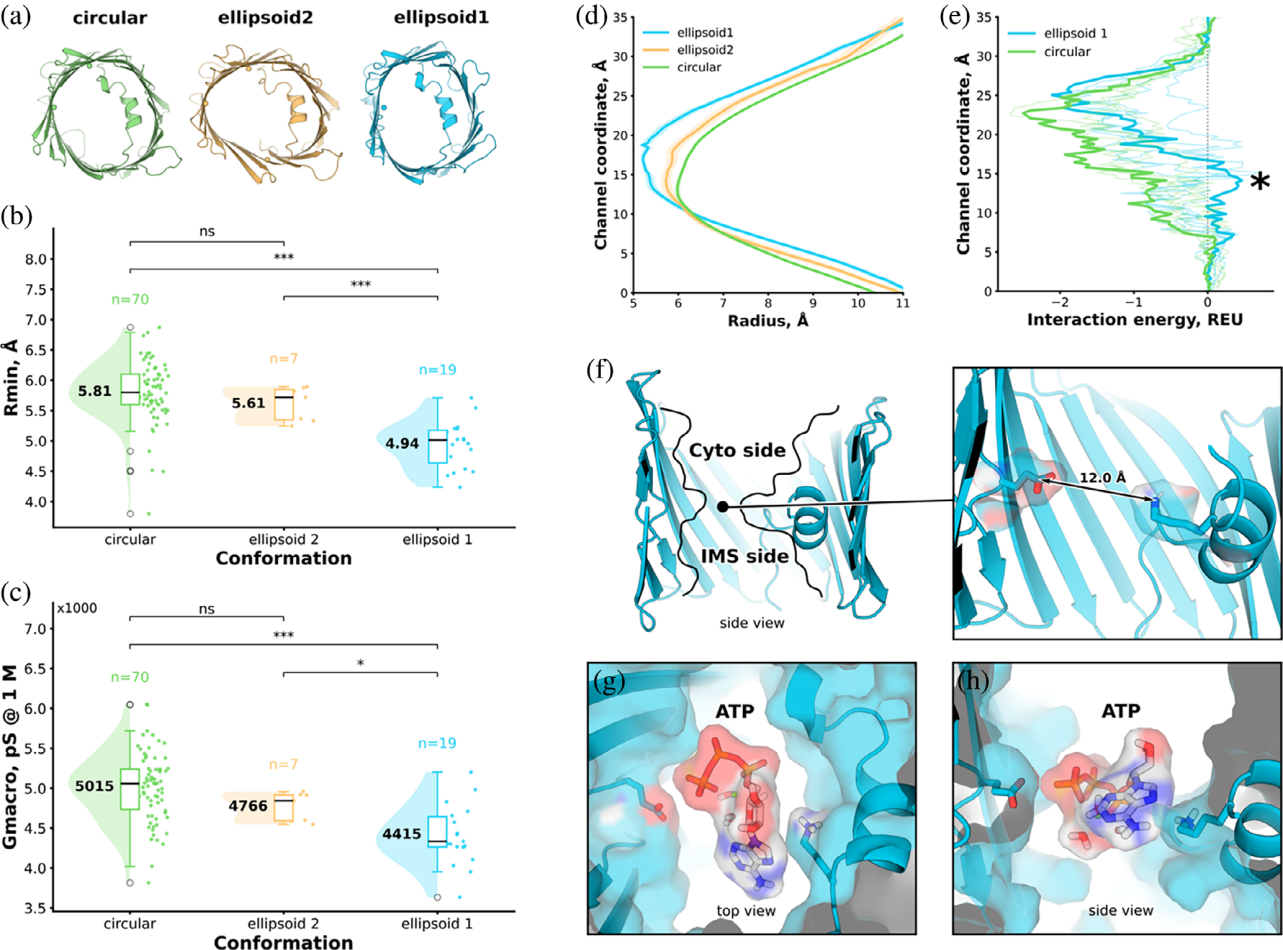
Geometric and functional characteristics of the VDAC1 conformations. (a) Representative structures of the three dominant conformational ensembles identified by DEER‐informed MD analysis: circular (green), ellipsoid 1 (blue), and ellipsoid 2 (orange). (b) Distribution of pore bottleneck radii (R_min) calculated using HOLE2 for each ensemble. Individual data points are representative frames; box plots indicate median and interquartile range, and half‐violin plots show the underlying distributions. The ellipsoid 1 ensemble exhibits a reduced bottleneck radius relative to the circular and ellipsoid 2 ensembles. (c) Estimated single‐channel conductance at 1 M salt concentration derived from pore geometry showing only modest but significant differences for the two ellipsoid conformations. (d) Axial pore‐radius profiles averaged over each ensemble, highlighting a pronounced mid‐pore constriction in the ellipsoid 1 conformation. (e) Predicted ATP interaction energy profiles along the channel axis, calculated using Rosetta LigandPathFinder. The ellipsoid 1 ensemble exhibits a positive free‐energy barrier in the bottleneck region (*), whereas the circular ensemble displays a continuous energetically favorable pathway. (f) Side view of the ellipsoid 1 conformations illustrating narrowing of the pore. (g), (h) Representative ATP poses within the ellipsoid 1 channel shown in top (g) and side (h) views, illustrating the bottleneck area.

To assess the functional implications of these conformations, we analyzed the pore bottleneck size, profile of the pore radius, and estimated channel conductance using the HOLE2 program (Smart et al., 1996). The ellipsoid 1 ensemble exhibited a reduced pore bottleneck radius relative to both the circular and ellipsoid 2 ensembles, with a pronounced constriction near the mid‐pore region (Figure 5b/d). However, estimated single‐channel conductance values derived from pore geometry differed only modestly between the different ensembles (Figure 5c), remaining substantially higher than conductance values typically associated with the “closed‐state” recorded by electrophysiological techniques (Choudhary et al., 2010, 2014). Because conductance estimates based on static structural models are sensitive to sampling and may not capture coupled rearrangements beyond the DEER‐informed regions, we interpret these values cautiously. We repeated this analysis using an extended set of frames collected from all five trajectories filtered using only distance criteria (SFig. 8). While we observe the same trend, the effect is blurred due to the larger contribution from spin‐label dynamics.

In contrast, ATP permeation simulations provided a more direct functional discriminator. Using Rosetta LigandPathFinder, ATP translocation through the ellipsoid 1 ensemble encountered a positive free‐energy barrier at the bottleneck, whereas the circular ensemble displayed a continuous energy funnel consistent with unhindered diffusion (Figure 5e). These results identify the ellipsoid 1 conformation as gating‐competent, capable of restricting metabolite permeation, even though it does not fully recapitulate a low‐conductance closed state observed electrophysiologically.

## 3 DISCUSSION

This study provides new insight into the structural dynamics of the VDAC, a key MOM protein essential for cellular homeostasis. Although *β*‐barrel membrane proteins are often regarded as structurally rigid, our combined experimental and computational data reveal that VDAC1 exhibits intrinsic conformational flexibility, consistent with earlier observations from simulation and spectroscopy studies (Najbauer et al., 2022; Choudhary et al., 2010; Ngo et al., 2022; Teijido et al., 2012; Zachariae et al., 2012). VDAC's unusual 19‐stranded *β*‐barrel topology may provide a unique balance between global stability and localized flexibility, enabling regulated gating transitions while preserving overall structural integrity.

By integrating atomistic MD simulations with DEER analysis under hydrostatic pressure, we identify reversible elliptical deformation of the *β*‐barrel as a defining structural feature associated with channel closure. Mid‐pore reporters (*α*2–*β*13^mid^, *β*1^mid^–*β*9^mid^) exhibited robust, pressure‐dependent distance shifts that are consistent with asymmetric expansion and compaction along an axis parallel to the N‐terminal segment. These observations indicate that VDAC gating involves coordinated barrel rearrangements rather than global unfolding.

In contrast to earlier models proposing large‐scale displacement or extrusion of the N‐terminal helix into the cytosol (Casadio et al., 2002; Colombini, 1989), we observed no evidence for such movements, even at 3 kbar hydrostatic pressure. Helix extrusion would be expected to produce pronounced increase in hydration and distance distributions, which were not detected. Recent work by Daniilidis et al. demonstrates N‐terminal displacement under conditions that promote oligomerization or strong confinement, including the use of small nanodiscs that mimic an oligomeric state (Daniilidis et al., 2025). In contrast, under more native‐like conditions—specifically monomeric VDAC reconstituted in larger nanodiscs—the N‐terminal helix remains positioned within the pore. Our results, obtained in monomeric nanodiscs, do not support large‐scale helix expulsion under these conditions. Moreover, our findings are consistent with prior crosslinking studies demonstrating that tethering the N‐terminus to the barrel wall—such as cysteine crosslinks between V17–A205 or L10–A170—does not abolish channel gating. Together, these results argue against models in which gating is driven primarily by wholesale N‐terminal displacement. Instead, our data support a mechanism centered on *β*‐strand rearrangements involving *β*1 and its structural continuation into *β*19 and *β*2–*β*5, producing an elliptical constriction that restricts metabolite translocation while maintaining intrapore positioning of the N‐terminal helix.

We further considered whether barrel rearrangements might arise from disruption of salt bridges, as prior work showed that acidification of the cytosolic side of VDAC1 alters gating behavior, likely by weakening electrostatic interactions. To test this possibility, we collected DEER measurements at pH 3.6 under atmospheric pressure. No significant changes in helix positioning were observed (SFig. 6), indicating that hydrostatic pressure–induced conformational changes cannot be mimicked by simple acidification. These findings suggest that the observed pressure‐driven barrel deformation arises primarily from side‐chain repacking, rather than from increased hydration or breakage of salt bridges.

In summary, we propose that VDAC gating occurs through a reversible elliptical deformation of the *β*‐barrel rather than by N‐terminal ejection. This mechanism accommodates regulation by diverse physical stimuli—including voltage, pressure, and membrane environment—while preserving the energetic efficiency of a compact *β*‐barrel fold. More broadly, our findings highlight *β*‐barrel plasticity as an under‐recognized regulatory principle in mitochondrial channels. By responding dynamically to metabolic cues and stress‐related perturbations, VDAC functions as a responsive mitochondrial gatekeeper, integrating bioenergetic demand with mitochondrial function.

## 4 METHODS

### 4.1 Molecular dynamics simulations

All‐atom MD simulations were initiated from configurations extracted from previously published Martini coarse‐grained (CG) simulations (Lafargue et al., 2025). These consisted of three independent replicas of membrane systems with identical composition, comprising VDAC1 in a 2:1 POPC:POPE mixture. Two snapshots per system (at 10 and 20 μs) were selected to increase sampling, yielding six replicas derived from independently built systems. This approach captures both stochastic variability (initial velocities) and differences in initial coordinates, including membrane packing and local protein–lipid environments, providing a stringent assessment of reproducibility. From these CG systems, the lipid configurations were retained and transformed into an all‐atom system using the backward procedure (Wassenaar et al., 2014). To maintain high‐resolution quality for the protein, the corresponding “backwarded structure” was replaced by a fresh all‐atom model previously derived from the experimental mouse hVDAC1 structure available at 2.3 Å resolution (PDB ID: 3EMN) (Ujwal et al., 2008).

The systems were resized around VDAC to 9.7 × 9.7 nm in the *xy* plane, with corresponding new periodic boundary conditions. The systems were relaxed to an equilibrium state using a series of energy minimization and constrained MD procedures described in the backward paper. The simulations were run using GROMACS software (Abraham et al., 2015) with the CHARMM36m force field with CMAP corrections (Huang et al., 2017).

The thermalization and equilibration process was developed over a six‐stage procedure where distinct position restraints were sequentially released. The first equilibration run applied restraints of 4000, 2000, and 1000 kJ/mol/nm^2^ to the protein backbone, side chains, and lipid head groups, respectively. In the final equilibration run, only small restraints (50 kJ/mol/nm^2^) remained on the atoms of the protein backbone. The system was heated to 303.15 K during the first run and controlled by a Berendsen thermostat. Pressure control was set using a Berendsen barostat at 1.0 bar in the second stage, employing semi‐isotropic control (*τ*_*p*_ = 5 ps and compressibility of 4.5 × 10^−5^ in the membrane plane and its normal direction). The first three equilibration processes ran over 125 ps, while 500 ps were used for the last three. The time step was increased from 1 to 2 fs in the fourth stage, and bond lengths involving hydrogen atoms were maintained constant using the LINCS algorithm throughout the simulations.

For the production period, which lasted over 500 ns, the Berendsen thermal and pressure control algorithms were replaced by the Nosé–Hoover thermostat and Parrinello–Rahman barostat schemes respectively. The remaining position restraints on the protein backbone were removed. The simulations were run on the supercomputer facilities provided by the GENCI at the TGCC (CEA)—Saclay France using GROMACS v2018.

### 4.2 Protein purification

mVDAC1 was produced in *Escherichia coli* and refolded from inclusion bodies. A detailed protocol is available in Dearden et al. (2024). Briefly, M15 *E. coli* cells were used to express mVDAC1 in inclusion bodies. The protein was isolated using a two‐step purification process: solubilized inclusion bodies were purified with a Talon metal affinity column, and the eluted protein was refolded using LDAO. The refolded protein was centrifuged at 200,000 × *g* for 45 min, and DTT was removed using a PD10 desalting column, with buffer exchanged to 20 mM Hepes (pH 7.0), 150 mM NaCl, and 0.1% LDAO to facilitate labeling. The protein was then incubated with a 1:1 molar ratio of the spin label reagent 1‐oxyl‐2,2,5,5‐tetramethylpyrroline‐3‐methyl methanethiosulfonate (MTSSL) for 30 min at 4°C to generate the R1 side chain. The protein was concentrated using an Amicon Ultra‐50k (Millipore) and subjected to size exclusion chromatography (Superdex 200; GE Healthcare) in 150 mM NaCl, 20 mM Tris·HCl (pH 8.0), and 0.1% LDAO to remove excess spin label and misfolded proteins. 20% glycerol‐D8 was added to the sample. All samples for the same mutants were concentration‐normalized (80–100 μM, depending on the mutant) to compare modulation depths and distance distributions.

### 4.3 Reconstitution into nanodiscs

Labeled VDAC was mixed with lipid mix 25 mM Egg PC (avanti lipids) in 50 mM sodium cholate, and membrane scaffold protein without a His‐tag (MSP1D1(−)). We used an 8 times excess of ND/VDAC to make sure we have monomeric VDAC per nanodiscs. The molar ratio of VDAC1/MSP1D1(−)/lipid was 1:16:800 in buffer 20 mM Tris pH 8.5 (to avoid dimerization; Bergdoll et al., 2018), 150 mM NaCl. The mixture was incubated for 60 min at 4°C, and nanodisc assembly was initiated by adding 60% (w/v) Bio‐Beads SM‐2 (Bio‐Rad), followed by incubation for 4–16 h at 4°C with rotation. Assembled nanodiscs containing His‐tagged VDAC protein were isolated by binding to Ni‐NTA resin equilibrated in buffer 20 mM Tris pH 8, 150 mM NaCl and washed with 10 column volumes of buffer to remove empty nanodiscs. Nanodiscs containing VDAC were eluted with 20 mM Tris pH 8, 150 mM NaCl, 300 mM imidazole, concentrated using an Amicon Ultra‐50k (Millipore) and subjected to size exclusion chromatography (column Superdex 200, 10/300; GE Healthcare) in buffer 20 mM Tris‐Cl, pH 8, 150 mM NaCl. 20% glycerol‐D8 was added to the sample. All samples for the same mutants were concentration‐normalized (80–100 μM, depending on the mutant) to compare modulation depths and distance distributions.

### 4.4 DEER experiment

4‐Pulse DEER experiments were conducted at 50 K on a Q‐band upgraded ElexSys 580 (Bruker, Germany) equipped with an arbitrary waveform generator, a 150 W traveling wave tube amplifier, and a QT‐2 probehead (Bruker, Germany) using the standard dead time free sequence (Pannier et al., 2000). Pulse lengths were optimized using echo nutation experiments and set to 16–20 ns and 32–40 ns for pi/2 and pi‐pulses, respectively. A 100 ns 50 MHz‐chirp pulse was applied as a pump pulse 70 MHz above the observer frequency.

Dipolar evolutions were recorded with 16 ns time resolution and analyzed model‐free in LongDistances v.1112 (Altenbach, 2021). The smoothness parameter was determined by L‐curve criterion. Standard deviations for distances were determined using the error analysis implementation in LongDistances using standard parameters and the inherited model‐free parameters.

### 4.5 Simulation of DEER distance distributions

Five MD trajectories (500 ns each, 1000 frames per trajectory) were used as input for the analysis. DEER distance distributions were simulated for residue pairs 16/100 and 28/141, selected because experimental measurements at 0 bar and 3 kbar show clear differences in the channel interior (Figure 3). At 3 kbar, the 16/100 distribution develops a shoulder in shorter distances area absent under normal conditions, whereas the 28/141 distribution exhibits a broadened tail toward longer distances.

For each frame of every trajectory, DEER distance distributions were simulated using the chiLife (v1.1.6) and MDAnalysis (v2.10.0) Python libraries (Michaud‐Agrawal et al., 2011; Tessmer & Stoll, 2023). MDAnalysis was used to iterate through the MD trajectories, while chiLife computed the DEER distribution for each frame. Ensembles of covalently attached spin labels were modeled using the rotamer library approach implemented in chiLife, employing the R1 label used in the original experiments (Tessmer & Stoll, 2023). Each per‐frame distance distribution was normalized to unity, as were the reference experimental DEER distributions.

In addition, DEER distance distributions were averaged over all frames for each trajectory, and the standard error of the mean (SEM) was calculated (SFig. 7). These averaged simulated distributions were compared with the experimental data to assess which trajectories reproduce the pressure‐dependent trends, namely the emergence of shorter distances for the 16/100 pair and longer distances for the 28/141 pair. Based on the comparison of distributions, trajectories T1, T2, and T4 were used for subsequent analysis. All the code is available on Zenodo repository.

### 4.6 Extraction of peak of simulated DEER distribution

To extract a single characteristic peak from each simulated DEER distribution, each per‐frame distribution was first smoothed with a one‐dimensional Gaussian filter (σ = 2; scipy.ndimage.gaussian_filter1d, SciPy v1.16.3) (Virtanen et al., 2020). This step reduces small fluctuations and noise that can create artificial minor peaks. Peaks were then identified using scipy.signal.find_peaks, and the peak with the highest intensity in the smoothed distribution was selected as the main peak for that frame.

### 4.7 DEER‐based filtering of MD simulation frames

To identify VDAC1 structures with a barrel shape deviating from the predominantly circular conformation observed under normal conditions (PDB: 3EMN), we applied a DEER‐based filtering procedure. Frames showing extreme positions of the main peak in the simulated DEER distributions were selected, capturing contributions from both protein backbone and spin label dynamics.

Two criteria were defined using the previously identified main peak of the per‐frame simulated DEER distributions:

1. d(16/100)≤ 22 Å and d(28/141)≥18 Å
2. d(16/100) ≥26 Å and d(28/141)≤ 17 Å

Frames satisfying criterion 1 were assigned to cluster 1, those satisfying criterion 2 to cluster 2, and frames meeting neither criterion were left unassigned. The selected frame numbers were written to GROMACS index (.ndx) files and used to extract the corresponding structures from trajectories T1, T2, and T4 (Abraham et al., 2015). This yielded six filtered trajectory sets in total. Cluster 1 contains frames in which the VDAC barrel is compressed along the 16/100 direction; cluster 2 includes near‐circular or 28/141‐compressed structures. Trajectories T1 and T4 contributed a larger fraction of frames to cluster 2, while T2 showed the opposite trend.

### 4.8 CA distance‐based filtering of MD simulation frames

The R1 spin label used in the DEER experiments is highly dynamic, making it difficult to separate the dynamics of protein backbone and spin labels (Polyhach et al., 2011; Sezer et al., 2008) in the computational analysis. To isolate the contribution of the dynamics of protein backbone, we applied an additional filtering step based solely on C*α*/C*α* distances.

For each frame of trajectories T1, T2, and T4, the Euclidean distance between C*α* atoms of residue pairs 16/100 and 28/141 was computed directly from trajectory coordinates using MDAnalysis. In T1, data points cluster near the center of the plot, consistent with a predominantly circular barrel geometry matching the x‐ray structure (PDB: 3EMN). T2 shows a shift toward shorter 16/100 and longer 28/141 distances, indicating compression along the 16/100 direction. T4 displays a tail toward longer 16/100 and shorter 28/141 distances, reflecting occasional compression along the 28/141 direction.

Three conformational classes were defined by the following C*α*/Cα distance thresholds:

1. d(16/100)≤ 24 Å and d(28/141) ≥35 Å–16/100‐compressed
2. d(16/100) ≥28 Å and d(28/141)≤ 31 Å–28/141‐compressed
3. 25 Å≤ d(16/100) ≤ 27 Å and 32 Å≤ d(28/141)≤34 Å–native‐like.

Criterion 1 was applied first; frames not satisfying it were tested for criterion 2; remaining frames were tested for criterion 3. Frames meeting none of the criteria were left unassigned.

To combine information from both filtering approaches, we retained frames passing both criteria simultaneously. The first approach is DEER‐based and is sensitive to both protein backbone and spin label dynamics. The second relies solely on C*α*–C*α* distances and therefore isolates the protein backbone contribution. Three final conformational groups were defined from these intersections:

1. Ellipsoid 1: DEER cluster 1 ∩ C*α* cluster 1 (Ellipsoid 1, frames from T2)
2. Ellipsoid 2: DEER cluster 2 ∩ C*α* cluster 2 (Ellipsoid 2, frames from T4)
3. Circular: DEER cluster 2 ∩ C*α* cluster 3 (Circular, frames from T1).

The Ellipsoid 1 frames come from T2, which demonstrates the same trend in the averaged simulated DEER distributions as the experimental data recorded at 3 kbar. This conformation therefore represents a geometry appearing, but not necessarily predominant, at elevated pressure. The Ellipsoid 2 and Circular frames come from T4 and T1, respectively, both of which reproduce the peak positions observed experimentally at 0 bar. These two conformations thus represent geometries present under normal conditions, with Circular being the predominant state. The three conformational groups were used for all subsequent structural analyses, including pore geometry (HOLE2) and ATP transport energy calculations (Rosetta LigandPathFinder).

### 4.9 Analysis of the VDAC1 pore geometry with HOLE2

To characterize the VDAC pore geometry, we used the HOLE2 program (Smart et al., 1996), which calculates the pore radius profile along the channel axis, the minimum pore radius (Rmin), and the estimated macroscopic conductance (Gmacro at 1 M KCl). HOLE2 was applied to every frame from each of the three conformational groups (circular, ellipsoid 1, and ellipsoid 2) using an identical setup.

Atomic radii were assigned using the simple AMBER van der Waals radius set provided with HOLE2. Before analysis, all frames were aligned to a common reference using the backbone heavy atoms of the *β*‐barrel in MDAnalysis to ensure a consistent starting orientation. The channel axis was defined along the Z direction, and the starting point for the pore search was set to (39.864, 49.011, 41.263) Å, corresponding to the pore center. A spherical probe was used in all calculations.

Rmin and Gmacro values were visualized as raincloud plots (Figure 5b,c). Differences between groups were evaluated using the two‐sided Mann–Whitney U test with Holm correction for multiple comparisons (scipy.stats.mannwhitneyu and statsmodels.stats.multitest.multipletests). Pore radius profiles were interpolated onto a common grid of 500 points spanning the full channel length using linear interpolation, with values outside the sampled region set to NaN. Mean profiles and standard errors of the mean were then calculated for each conformational group (Figure 5d). The Python code used for analysis and plotting, together with HOLE2 input files, is available in the Zenodo repository.

### 4.10 Evaluation of the energy of ATP transport with Rosetta LigandPathFinder

To estimate whether ATP transport is hindered in the ellipsoid 1 conformation compared to the native‐like circular conformation, we used the Rosetta LigandPathFinder toolkit. One representative structure from each conformational ensemble was used as input. For ellipsoid 1, we selected the structure with the narrowest bottleneck, defined as the minimum diameter of 12.0 Å between closest heavy atoms (Figure 5f). For the circular conformation, we selected the structure closest to the x‐ray structure (PDB: 3EMN) by RMSD of β‐barrel backbone heavy atoms.

For Rosetta LigandPathFinder, we used a complex of ATP^4−^, Mg^2+^ and two water molecules as the ligand. The initial coordinates were taken from DFT calculations, and the library of 275 conformers was created with BCL by employing RMSD clustering at 0.25 Å level (Mendenhall et al., 2021; Mudryk et al., 2020). Guide paths for tunnel exploration were initialized as straight lines going through centers of mass of two structures. Standard parameters were used (full inputs provided in the SI), with the step size of 0.33 Å resulting in trajectories with 153 frames. RMSD restraint for ligand conformations between frames had a standard deviation of 1.0 Å.

For each representative structure, Rosetta LigandPathFinder simulated 100 independent ATP transport trajectories along the VDAC channel axis. Each trajectory was represented as a sequence of ATP coordinate sets and loaded using the ProDy Python library (v2.6.1) (Bakan et al., 2011). Since individual trajectories may differ from one another, we assessed their variability by computing pairwise distances between all trajectory pairs. Each pairwise distance was defined as the uniform‐weight mean of per‐frame RMSDs. Trajectories were then grouped using Affinity Propagation clustering applied to this precomputed distance matrix. For each resulting cluster, the mean interaction energy profile was calculated across all member trajectories. A cluster was classified as dominant if its size exceeded 95% of the size of the largest cluster. The mean energy profile of the dominant cluster was used as the representative profile for each conformational group. Interaction energy profiles were plotted as a function of the channel coordinate (Figure 5e). The positioning of ATP in the bottleneck region of the ellipsoid 1 conformation is illustrated in Figure 5g,h. Scripts for the analysis and visualization of Rosetta LigandPathFinder results are available in the Zenodo repository.

### 4.11 Additional statistics based on C*α*/C*α*‐distances‐only filtered frames

To assess pore geometry across a broader and more diverse set of structures, we repeated the HOLE2 analysis on an extended set of frames. In contrast to the combined DEER–C*α*/C*α* Distance approach described above, no DEER‐based filtering was applied here. Frames were selected solely based on C*α*/C*α* distance thresholds, and all five trajectories (T1–T5) were included. Each frame was independently assigned to one of three conformational classes if it satisfied the corresponding criteria:

1. d(16/100) ≤24 Å and d(28/141) ≥35 Å–Ellipsoid 1
2. d(16/100) ≥28 Å and d(28/141)≤ 31 Å–Ellipsoid 2
3. 25 Å≤ d(16/100)≤ 27 Å and 32 Å≤ d(28/141)≤ 34 Å Circular

This classification yielded *n* = 75 frames for Ellipsoid 1, *n* = 131 for Ellipsoid 2, and *n* = 2026 for Circular, with frames originating from all five trajectories. For each classified frame, HOLE2 was run using the same setup described above. Rmin and Gmacro values were extracted from the HOLE2 output files. Mean pore‐radius profiles and standard errors of the mean were calculated within each conformational class. Rmin and Gmacro distributions were visualized as raincloud plots (SFig. 8a,b), and between‐group differences were evaluated using the two‐sided Mann–Whitney U test with Holm correction for multiple comparisons. Pore radius profiles were read from precomputed CSV files and interpolated onto a common grid of 500 points, as described above (Figure 5).

The results obtained with this distance‐only classification (T1–T5) are qualitatively and quantitatively similar to those from the combined DEER/distance‐based filtering applied to T1, T2, and T4 (Figure 5 and SFig. 8). However, the DEER‐assisted approach provides clearer and more accurate discrimination between VDAC conformations. The Python code used for this analysis is available in the Zenodo repository.

## AUTHOR CONTRIBUTIONS

**M. Elgeti:** Investigation; funding acquisition; writing – original draft; writing – review and editing; methodology; formal analysis; data curation. **L. Bergdoll:** Conceptualization; investigation; writing – original draft; formal analysis. **A. Zlobin:** Methodology; data curation; software. **J. P. Duneau:** Methodology; software; data curation. **J. Belyaeva:** Investigation; methodology; formal analysis; data curation. **J. Abramson:** Conceptualization; funding acquisition; writing – original draft; writing – review and editing; project administration; supervision. **W. Hubbell:** Supervision; funding acquisition.

## FUNDING INFORMATION

This work was supported by NIH grant R35GM135175 (JA), R01GM078844 and the Jules Stein Professorship Endowment (to WLH) and R01GM137081 (to ME). This work was performed using HPC resources from GENCI–IDRIS (Grant 2021‐A0110707044 2022 and 2023‐AD010707044R1).

## CONFLICT OF INTEREST STATEMENT

The authors declare no conflicts of interest.

## Supporting information


**Supplementary Fig. 1:** Motion of VDAC1 in all‐atom simulations. Six structures of VDAC1, spanning 500 ns molecular dynamics simulations, are color‐coded based on the computed root mean square fluctuation (RMSF). These structures are shown from four perspectives. (B) Root mean square deviations (RMSD) of the backbone from the six 500 ns simulations.
**SFig. 2.** Four‐pulse DEER dipolar evolution traces for the double‐cysteine mutants shown in Figure 2. VDAC samples recorded in LDAO detergent (gray) and lipid nanodiscs (ND; colored traces). Solid lines represent six‐Gaussian model‐based fits to the experimental dipolar evolution data. Cytosolic (cyt), mid barrel (mid), and intermembrane space (ims) labeling pairs are indicated in each panel. All traces are background‐corrected and normalized as *V*(*t*)/*V*(0). Time is shown in microseconds (μs).
**SFig. 3.** High pressure enhances intermolecular contributions in detergent‐solubilized VDAC. (A) Four‐pulse DEER dipolar evolution traces of VDAC1 *β*7^ims^–*β*17^ims^ (119R1–250R1) in LDAO detergent at pH 8 (red), under 3 kbar hydrostatic pressure (blue), and under 3 kbar in the presence of excess unlabeled VDAC to dilute intermolecular spin–spin interactions (green). (B) Corresponding distance distributions obtained from Tikhonov regularization. (C) Modulation depth values under each condition. Application of 3 kbar increases modulation depth and enhances longer‐distance components, consistent with pressure‐induced oligomerization. Addition of unlabeled VDAC reduces modulation depth under pressure, indicating suppression of intermolecular dipolar contributions.
**SFig. 4.** Reversibility of pressure‐induced distance changes. Distance distributions for the double‐cysteine mutants shown in Figure 3 following decompression to atmospheric pressure (“return”). Colored traces represent the distance distributions after pressure release, overlaid with the corresponding atmospheric‐pressure distributions (gray) for comparison. For all labeling positions, the post‐pressure profiles closely resemble the initial atmospheric state, indicating that the pressure‐induced conformational changes are largely reversible.
**SFig. 5.** Workflow for DEER‐informed analysis of MD trajectories. (a) Five independent, unbiased MD trajectories (T1–T5; 1000 frames each) were analyzed to identify conformational states consistent with experimental DEER data. (b) For each trajectory, spin labels were attached in silico and DEER distance distributions were simulated for every frame using chiLife. Simulated DEER traces were averaged across each trajectory and compared with experimental distributions obtained at ambient pressure (0 bar) and under 3 kbar. A DEER‐based filtering procedure was applied to select trajectories and frames reproducing the experimentally observed pressure dependent shifts. Extreme cases corresponding to ambient‐like (Cluster 1) and pressure‐stabilized (Cluster 2) distance distributions were identified and the corresponding simulation frames were extracted. (c) A subsequent distance‐based filtering and clustering step yielded representative structural models corresponding to circular (ambient/open‐like) and elliptical (pressure‐consistent) conformations. These representative ensembles were used for downstream structural analysis, including pore geometry and channel radius profiling.
**SFig. 6.** Acidification does not reproduce pressure‐induced conformational changes. Left: Four‐pulse DEER dipolar evolution traces (gray dots) and corresponding six‐Gaussian model based fits (solid lines) for VDAC1 16R1–100R1 in lipid nanodiscs at pH 8 (black) and pH 3.6 (red) under atmospheric pressure. Right: Corresponding distance distributions. No significant shift in the primary distance peak is observed upon acidification, indicating that lowering pH does not mimic the conformational changes induced by hydrostatic pressure.
**SFig. 7.** Averaged simulated DEER distance distributions across independent MD trajectories. Simulated DEER distance distributions for the d(16/100) spin‐label pair averaged over all frames within each independent MD trajectory (T1–T5). Shaded regions represent the standard error of the mean (SEM). Corresponding averaged simulated DEER distance distributions for the d(28/141) spin‐label pair for each trajectory, with SEM indicated. Comparison of the trajectory‐averaged simulated distributions with experimental DEER data was used to assess which simulations reproduce the pressure‐dependent trends—specifically, shorter distances for d(16/100) and longer distances for d(28/141). Based on this analysis, trajectories T1, T2, and T4 were selected for subsequent ensemble characterization.
**SFig. S8.** Structural and functional comparison of representative DEER‐consistent conformational ensembles. (a) Distribution of pore bottleneck radii (R_min) calculated using HOLE2 for the circular (green), ellipsoid 2 (orange), and ellipsoid 1 (blue) ensembles derived from the selected MD trajectories (T1, T2, and T4). Individual points represent analyzed frames; box plots indicate median and interquartile range, and violin plots show the underlying distributions. Statistical significance between ensembles is indicated (ns, not significant; ***p <0.001). (b) Estimated single‐channel conductance (G_macro) at 1 M salt concentration calculated from pore geometry for the same ensembles. Although differences are observed, conductance values remain substantially higher than those typically associated with electrophysiologically closed states. (c) Axial pore‐radius profiles averaged over each ensemble, illustrating a pronounced mid‐pore constriction in the ellipsoid 1 conformation relative to the circular and ellipsoid 2 states.
**SFig. S9.** Four‐pulse DEER dipolar evolution traces for the double‐cysteine mutants shown in Fig. 3. DEER dipolar evolution data (gray dots) and 6‐Gaussian model‐based fits (plain lines) for the mutants presented Fig. 3.

## Data Availability

Zenodo repository: The MD trajectories used in this study, together with the Python scripts for data analysis and plotting, and the outputs from HOLE2 and Rosetta LigandPathFinder, are available in the Zenodo repository (DOI: https://doi.org/10.5281/zenodo.18679735).

## References

[pro70677-bib-0001] Abraham MJ , Murtola T , Schulz R , Páll S , Smith JC , Hess B , et al. GROMACS: high performance molecular simulations through multi‐level parallelism from laptops to supercomputers. SoftwareX. 2015;1‐2:19–25.

[pro70677-bib-0002] Akasaka K . Highly fluctuating protein structures revealed by variable‐pressure nuclear magnetic resonance. Biochemistry. 2003;42:10875–10885. 12974621 10.1021/bi034722p

[pro70677-bib-0003] Altenbach C . LongDistances—a program to analyze DEER data. EPR Newletter. 2021;31:12–13.

[pro70677-bib-0004] Bakan A , Meireles LM , Bahar I . ProDy: protein dynamics inferred from theory and experiments. Bioinformatics. 2011;27:1575–1577. 21471012 10.1093/bioinformatics/btr168 PMC3102222

[pro70677-bib-0005] Bayrhuber M , Meins T , Habeck M , Becker S , Giller K , Villinger S , et al. Structure of the human voltage‐dependent anion channel. Proc Natl Acad Sci USA. 2008;105:15370–15375. 18832158 10.1073/pnas.0808115105 PMC2557026

[pro70677-bib-0006] Belyaeva J , Elgeti M . Exploring protein structural ensembles: integration of sparse experimental data from electron paramagnetic resonance spectroscopy with molecular modeling methods. Elife. 2024;13:e99770. 39283059 10.7554/eLife.99770 PMC11405019

[pro70677-bib-0007] Bergdoll L , Grabe M , Abramson J . An assessment of how VDAC structures have impacted our understanding of their function. In: Rostovtseva TK , editor. Molecular basis for mitochondrial signaling. Cham: Springer International Publishing; 2017. p. 141–160.

[pro70677-bib-0008] Bergdoll LA , Lerch MT , Patrick JW , Belardo K , Altenbach C , Bisignano P , et al. Protonation state of glutamate 73 regulates the formation of a specific dimeric association of mVDAC1. Proc Natl Acad Sci USA. 2018;115:E172–E179. 29279396 10.1073/pnas.1715464115 PMC5777057

[pro70677-bib-0009] Casadio R , Jacoboni I , Messina A , de Pinto V . A 3D model of the voltage‐dependent anion channel (VDAQ). FEBS Lett. 2002;520:1–7. 12044860 10.1016/s0014-5793(02)02758-8

[pro70677-bib-0010] Choudhary OP , Paz A , Adelman JL , Colletier JP , Abramson J , Grabe M . Structure‐guided simulations illuminate the mechanism of ATP transport through VDAC1. Nat Struct Mol Biol. 2014;21:626–632. 24908397 10.1038/nsmb.2841 PMC4157756

[pro70677-bib-0011] Choudhary OP , Ujwal R , Kowallis W , Coalson R , Abramson J , Grabe M . The electrostatics of VDAC: implications for selectivity and gating. J Mol Biol. 2010;396:580–592. 20005234 10.1016/j.jmb.2009.12.006 PMC3736979

[pro70677-bib-0012] Colombini M . Voltage gating in the mitochondrial channel, VDAC. J Membr Biol. 1989;111:103–111. 2482359 10.1007/BF01871775

[pro70677-bib-0013] Colombini M . VDAC: the channel at the interface between mitochondria and the cytosol. Mol Cell Biochem. 2004;256:107–115. 14977174 10.1023/b:mcbi.0000009862.17396.8d

[pro70677-bib-0014] Daniilidis M , Günsel U , Broutzakis G , Leitl KD , Janowski R , Fredriksson K , et al. Structural basis of apoptosis induction by the mitochondrial voltage‐dependent anion channel. Nat Commun. 2025;16:9481. 41145501 10.1038/s41467-025-65363-1 PMC12559223

[pro70677-bib-0015] Dearden GI , Ravishankar V , Sakata KT , Menon AK , Bergdoll L . Protocol for the production and reconstitution of VDAC1 for functional assays. STAR Protoc. 2024;5:103240. 39116198 10.1016/j.xpro.2024.103240 PMC11383923

[pro70677-bib-0017] Gonzalez‐DeWhitt KR , Ermolova N , Wang HK , Hekstra DR , Althoff T , Abramson J . Insights into VDAC gating: room‐temperature x‐ray Crystal structure of mVDAC‐1. Biomolecules. 2024;14:1203. 39456136 10.3390/biom14101203 PMC11505624

[pro70677-bib-0018] Heslop KA , Milesi V , Maldonado EN . VDAC modulation of cancer metabolism: advances and therapeutic challenges. Front Physiol. 2021;12:742839. 34658929 10.3389/fphys.2021.742839 PMC8511398

[pro70677-bib-0019] Hiller S , Garces RG , Malia TJ , Orekhov VY , Colombini M , Wagner G . Solution structure of the integral human membrane protein VDAC‐1 in detergent micelles. Science. 2008;321:1206–1210. 18755977 10.1126/science.1161302 PMC2579273

[pro70677-bib-0020] Huang J , Rauscher S , Nawrocki G , Ran T , Feig M , de Groot BL , et al. CHARMM36: an improved force field for folded and intrinsically disordered proteins. Biophys J. 2017;112:175a–176a. 10.1038/nmeth.4067 PMC5199616 27819658

[pro70677-bib-0021] Lafargue E , Duneau JP , Buzhinsky N , Ornelas P , Ortega A , Ravishankar V , et al. Membrane lipid composition modulates the organization of VDAC1, a mitochondrial gatekeeper. Commun Biol. 2025;8:936. 40527982 10.1038/s42003-025-08311-5 PMC12174345

[pro70677-bib-0022] Lemasters JJ , Holmuhamedov E . Voltage‐dependent anion channel (VDAC) as mitochondrial governator—thinking outside the box. Biochim Biophys Acta. 2006;1762:181–190. 16307870 10.1016/j.bbadis.2005.10.006

[pro70677-bib-0023] Lerch MT , Matt RA , Masureel M , Elgeti M , Kumar KK , Hilger D , et al. Viewing rare conformations of the beta(2) adrenergic receptor with pressure‐resolved DEER spectroscopy. Proc Natl Acad Sci USA. 2020;117:31824–31831. 33257561 10.1073/pnas.2013904117 PMC7749303

[pro70677-bib-0024] Lerch MT , Yang Z , Brooks EK , Hubbell WL . Mapping protein conformational heterogeneity under pressure with site‐directed spin labeling and double electron‐electron resonance. Proc Natl Acad Sci USA. 2014;111:E1201–E1210. 24707053 10.1073/pnas.1403179111 PMC3977274

[pro70677-bib-0025] Mendenhall J , Brown BP , Kothiwale S , Meiler J . BCL::conf: improved open‐source knowledge‐based conformation sampling using the crystallography open database. J Chem Inf Model. 2021;61:189–201. 33351632 10.1021/acs.jcim.0c01140 PMC8130828

[pro70677-bib-0026] Mertins B , Psakis G , Grosse W , Back KC , Salisowski A , Reiss P , et al. Flexibility of the N‐terminal mVDAC1 segment controls the channel's gating behavior. PLoS One. 2012;7:e47938. 23110136 10.1371/journal.pone.0047938 PMC3479125

[pro70677-bib-0027] Michaud‐Agrawal N , Denning EJ , Woolf TB , Beckstein O . MDAnalysis: a toolkit for the analysis of molecular dynamics simulations. J Comput Chem. 2011;32:2319–2327. 21500218 10.1002/jcc.21787 PMC3144279

[pro70677-bib-0028] Mudryk KD , Seidel R , Winter B , Wilkinson I . The electronic structure of the aqueous permanganate ion: aqueous‐phase energetics and molecular bonding studied using liquid jet photoelectron spectroscopy. Phys Chem Chem Phys. 2020;22:20311–20330. 32895669 10.1039/d0cp04033a

[pro70677-bib-0029] Najbauer EE , Tekwani Movellan K , Giller K , Benz R , Becker S , Griesinger C , et al. Structure and gating behavior of the human integral membrane protein VDAC1 in a lipid bilayer. J Am Chem Soc. 2022;144:2953–2967. 35164499 10.1021/jacs.1c09848 PMC8874904

[pro70677-bib-0030] Ngo VA , Queralt‐Martín M , Khan F , Bergdoll L , Abramson J , Bezrukov SM , et al. The single residue K12 governs the exceptional voltage sensitivity of mitochondrial voltage‐dependent anion channel gating. J Am Chem Soc. 2022;144:14564–14577. 35925797 10.1021/jacs.2c03316 PMC12293989

[pro70677-bib-0031] Pannier M , Veit S , Godt A , Jeschke G , Spiess HW . Dead‐time free measurement of dipole–dipole interactions between electron spins. J Magn Reson. 2000;142:331–340. 10648151 10.1006/jmre.1999.1944

[pro70677-bib-0032] Polyhach Y , Bordignon E , Jeschke G . Rotamer libraries of spin labelled cysteines for protein studies. Phys Chem Chem Phys. 2011;13:2356–2366. 21116569 10.1039/c0cp01865a

[pro70677-bib-0033] Qi Y , Lee J , Cheng X , Shen R , Islam SM , Roux B , et al. CHARMM‐GUI DEER facilitator for spin‐pair distance distribution calculations and preparation of restrained‐ensemble molecular dynamics simulations. J Comput Chem. 2020;41:415–420. 31329318 10.1002/jcc.26032

[pro70677-bib-0034] Queralt‐Martin M , Bergdoll L , Jacobs D , Bezrukov SM , Abramson J , Rostovtseva TK . Assessing the role of residue E73 and lipid headgroup charge in VDAC1 voltage gating. Biochim Biophys Acta Bioenerg. 2019;1860:22–29. 30412693 10.1016/j.bbabio.2018.11.001 PMC8283775

[pro70677-bib-0035] Rosencrans WM , Khuntia H , Ghahari Larimi M , Mahalakshmi R , Yu TY , Bezrukov SM , et al. Conformational plasticity of mitochondrial VDAC2 controls the kinetics of its interaction with cytosolic proteins. Sci Adv. 2025;11:eadv4410. 40267181 10.1126/sciadv.adv4410 PMC12017312

[pro70677-bib-0036] Rostovtseva T , Colombini M . ATP flux is controlled by a voltage‐gated channel from the mitochondrial outer membrane. J Biol Chem. 1996;271:28006–28008. 8910409 10.1074/jbc.271.45.28006

[pro70677-bib-0037] Rostovtseva T , Colombini M . VDAC channels mediate and gate the flow of ATP: implications for the regulation of mitochondrial function. Biophys J. 1997;72:1954–1962. 9129800 10.1016/S0006-3495(97)78841-6 PMC1184392

[pro70677-bib-0052] Rostovtseva TK , Kazemi N , Weinrich M , Bezrukov SM . Voltage gating of VDAC is regulated by nonlamellar lipids of mitochondrial membranes. J Biol Chem. 2006;281:37496–37506. 16990283 10.1074/jbc.M602548200

[pro70677-bib-0038] Rostovtseva TK , Bezrukov SM . VDAC regulation: role of cytosolic proteins and mitochondrial lipids. J Bioenerg Biomembr. 2008;40:163–170. 18654841 10.1007/s10863-008-9145-y PMC2671000

[pro70677-bib-0039] Sezer D , Freed JH , Roux B . Parametrization, molecular dynamics simulation, and calculation of electron spin resonance spectra of a nitroxide spin label on a polyalanine alpha‐helix. J Phys Chem B. 2008;112:5755–5767. 18412413 10.1021/jp711375x PMC2766176

[pro70677-bib-0040] Smart OS , Neduvelil JG , Wang X , Wallace BA , Sansom MS . HOLE: a program for the analysis of the pore dimensions of ion channel structural models. J Mol Graph. 1996;14:354–360, 376. 9195488 10.1016/s0263-7855(97)00009-x

[pro70677-bib-0041] Teijido O , Rappaport SM , Chamberlin A , Noskov SY , Aguilella VM , Rostovtseva TK , et al. Acidification asymmetrically affects voltage‐dependent anion channel implicating the involvement of salt bridges. J Biol Chem. 2014;289:23670–23682. 24962576 10.1074/jbc.M114.576314 PMC4156087

[pro70677-bib-0042] Teijido O , Ujwal R , Hillerdal CO , Kullman L , Rostovtseva TK , Abramson J . Affixing N‐terminal alpha‐helix to the wall of the voltage‐dependent anion channel does not prevent its voltage gating. J Biol Chem. 2012;287:11437–11445. 22275367 10.1074/jbc.M111.314229 PMC3322836

[pro70677-bib-0043] Tessmer MH , Stoll S . chiLife: an open‐source python package for in silico spin labeling and integrative protein modeling. PLoS Comput Biol. 2023;19:e1010834. 37000838 10.1371/journal.pcbi.1010834 PMC10096462

[pro70677-bib-0044] Ujwal R , Cascio D , Colletier JP , Faham S , Zhang J , Toro L , et al. The crystal structure of mouse VDAC1 at 2.3 a resolution reveals mechanistic insights into metabolite gating. Proc Natl Acad Sci USA. 2008;105:17742–17747. 18988731 10.1073/pnas.0809634105 PMC2584669

[pro70677-bib-0045] Varughese JT , Buchanan SK , Pitt AS . The role of voltage‐dependent Anion Channel in mitochondrial dysfunction and human disease. Cells. 2021;10:1737. 34359907 10.3390/cells10071737 PMC8305817

[pro70677-bib-0046] Verma A , Shteinfer‐Kuzmine A , Kamenetsky N , Pittala S , Paul A , Nahon Crystal E , et al. Targeting the overexpressed mitochondrial protein VDAC1 in a mouse model of Alzheimer's disease protects against mitochondrial dysfunction and mitigates brain pathology. Transl Neurodegener. 2022;11:58. 36578022 10.1186/s40035-022-00329-7 PMC9795455

[pro70677-bib-0047] Virtanen P , Oliphant TE , Haberland M , Reddy T , Cournapeau D , Burovski E , et al. SciPy 1.0: fundamental algorithms for scientific computing in Python. Nat Methods. 2020;17:261–272. 32015543 10.1038/s41592-019-0686-2 PMC7056644

[pro70677-bib-0048] Wassenaar TA , Pluhackova K , Bockmann RA , Marrink SJ , Tieleman DP . Going backward: a flexible geometric approach to reverse transformation from coarse grained to atomistic models. J Chem Theory Comput. 2014;10:676–690. 26580045 10.1021/ct400617g

[pro70677-bib-0049] Yuan S , Sun R , Shi H , Chapman NM , Hu H , Guy C , et al. VDAC2 loss elicits tumour destruction and inflammation for cancer therapy. Nature. 2025;640:1062–1071. 40108474 10.1038/s41586-025-08732-6 PMC12018455

[pro70677-bib-0050] Zachariae U , Schneider R , Briones R , Gattin Z , Demers JP , Giller K , et al. Beta‐barrel mobility underlies closure of the voltage‐dependent anion channel. Structure. 2012;20:1540–1549. 22841291 10.1016/j.str.2012.06.015 PMC5650048

[pro70677-bib-0051] Zeth K , Thein M . Porins in prokaryotes and eukaryotes: common themes and variations. Biochem J. 2010;431:13–22. 20836765 10.1042/BJ20100371

