# Supplementary material for: Elliptical β ‐barrel deformation underlies gating in VDAC1
Source: Protein Sci. 2026 Jun 19;35(7):e70677. doi: 10.1002/pro.70677 (PMC13281165; doi:10.1002/pro.70677)
Supplement: Supplementary file 1 — Supplementary Fig. 1: Motion of VDAC1 in all‐atom simulations. Six structures of VDAC1, spanning 500 ns molecular dynamics simulations, are color‐coded based on the computed root mean square fluctuation (RMSF). These structures are shown from four perspectives. (B) Root mean square deviations (RMSD) of the backbone from the six 500 ns simulations. SFig. 2. Four‐pulse DEER dipolar evolution traces for the double‐cysteine mutants shown in Figure 2. VDAC samples recorded in LDAO detergent (gray) and lipid nanodiscs (ND; colored traces). Solid lines represent six‐Gaussian model‐based fits to the experimental dipolar evolution data. Cytosolic (cyt), mid barrel (mid), and intermembrane space (ims) labeling pairs are indicated in each panel. All traces are background‐corrected and normalized as V(t)/V(0). Time is shown in microseconds (μs). SFig. 3. High pressure enhances intermolecular contributions in detergent‐solubilized VDAC. (A) Four‐pulse DEER dipolar evolution traces of VDAC1 β7ims–β17ims (119R1–250R1) in LDAO detergent at pH 8 (red), under 3 kbar hydrostatic pressure (blue), and under 3 kbar in the presence of excess unlabeled VDAC to dilute intermolecular spin–spin interactions (green). (B) Corresponding distance distributions obtained from Tikhonov regularization. (C) Modulation depth values under each condition. Application of 3 kbar increases modulation depth and enhances longer‐distance components, consistent with pressure‐induced oligomerization. Addition of unlabeled VDAC reduces modulation depth under pressure, indicating suppression of intermolecular dipolar contributions. SFig. 4. Reversibility of pressure‐induced distance changes. Distance distributions for the double‐cysteine mutants shown in Figure 3 following decompression to atmospheric pressure (“return”). Colored traces represent the distance distributions after pressure release, overlaid with the corresponding atmospheric‐pressure distributions (gray) for comparison. For all labeling positions, t [file PRO-35-e70677-s001.pdf]

Supplemental Information

Elliptical β-Barrel Deformation Underlies Gating in VDAC1

Bergdoll L[1*#], Elgeti M[2*#], Belyaeva J[2], Zlobin A[3], Duneau JP[1], Hubbell W[4], Abramson J[5#]

[1]Laboratoire d'Ingénierie des Systèmes Macromoléculaires, CNRS; UMR 7255 - Aix Marseille Université, 31 Chemin Joseph Aiguier; 13402 Marseille, France
[2]Institute for Drug Discovery and Institute for Medical Physics and Biophysics, University of Leipzig Medical School; Härtelstr. 16-18; 04107 Leipzig, Germany
[3]Institute for Drug Discovery and Institute for Medical Physics and Biophysics, Leipzig University Medical School; Liebigstraße 21; 04103 Leipzig, Germany
[4]Stein Eye Institute, David Geffen School of Medicine; University of California, Los Angeles; Los Angeles, CA 90095, USA
[5]Department of Physiology, David Geffen School of Medicine; University of California, Los Angeles; Los Angeles, CA 90095, USA
* : equal contribution

# : to whom correspondence should be addressed

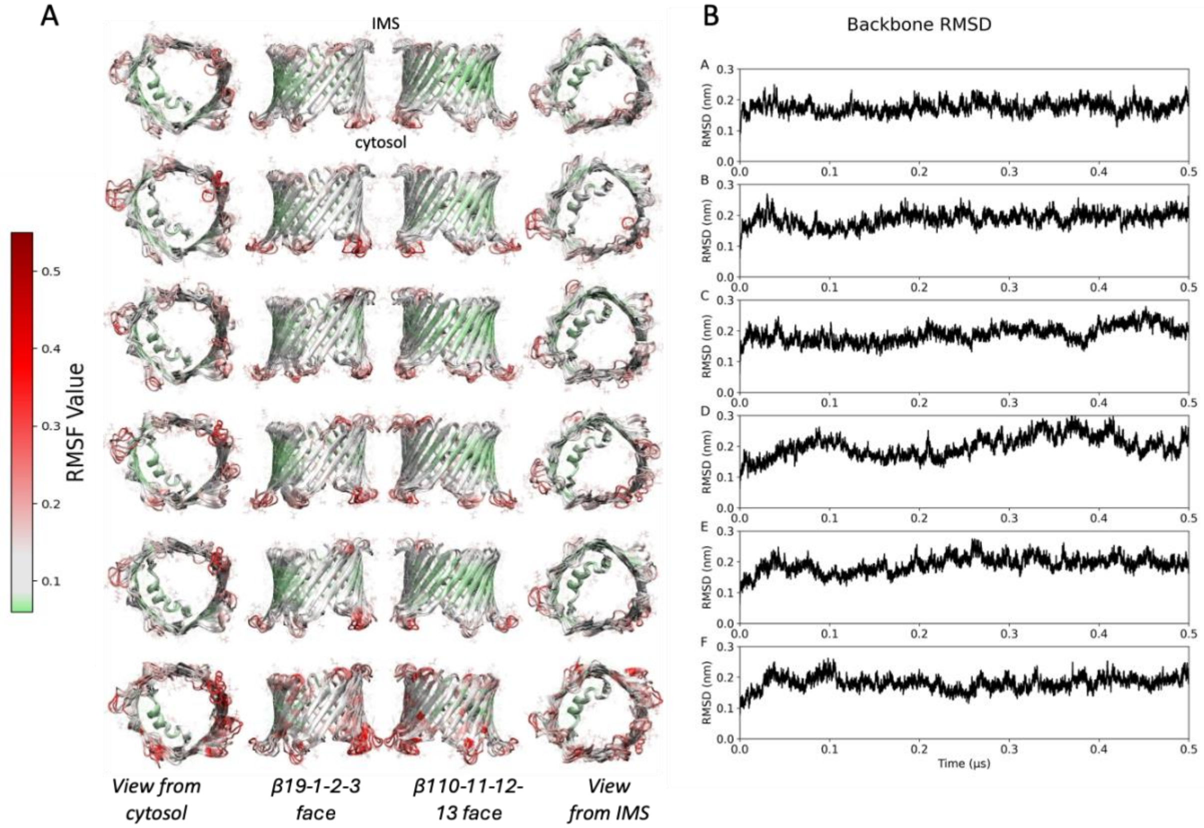

SFig. 1: Motion of VDAC1 in all-atom simulations. Six structures of VDAC1, spanning 500 ns molecular dynamics simulations, are color-coded based on the computed Root Mean Square Fluctuation (RMSF). These structures are shown from four perspectives. (B) Root Mean Square Deviations (RMSD) of the backbone from the six 500 ns simulations.

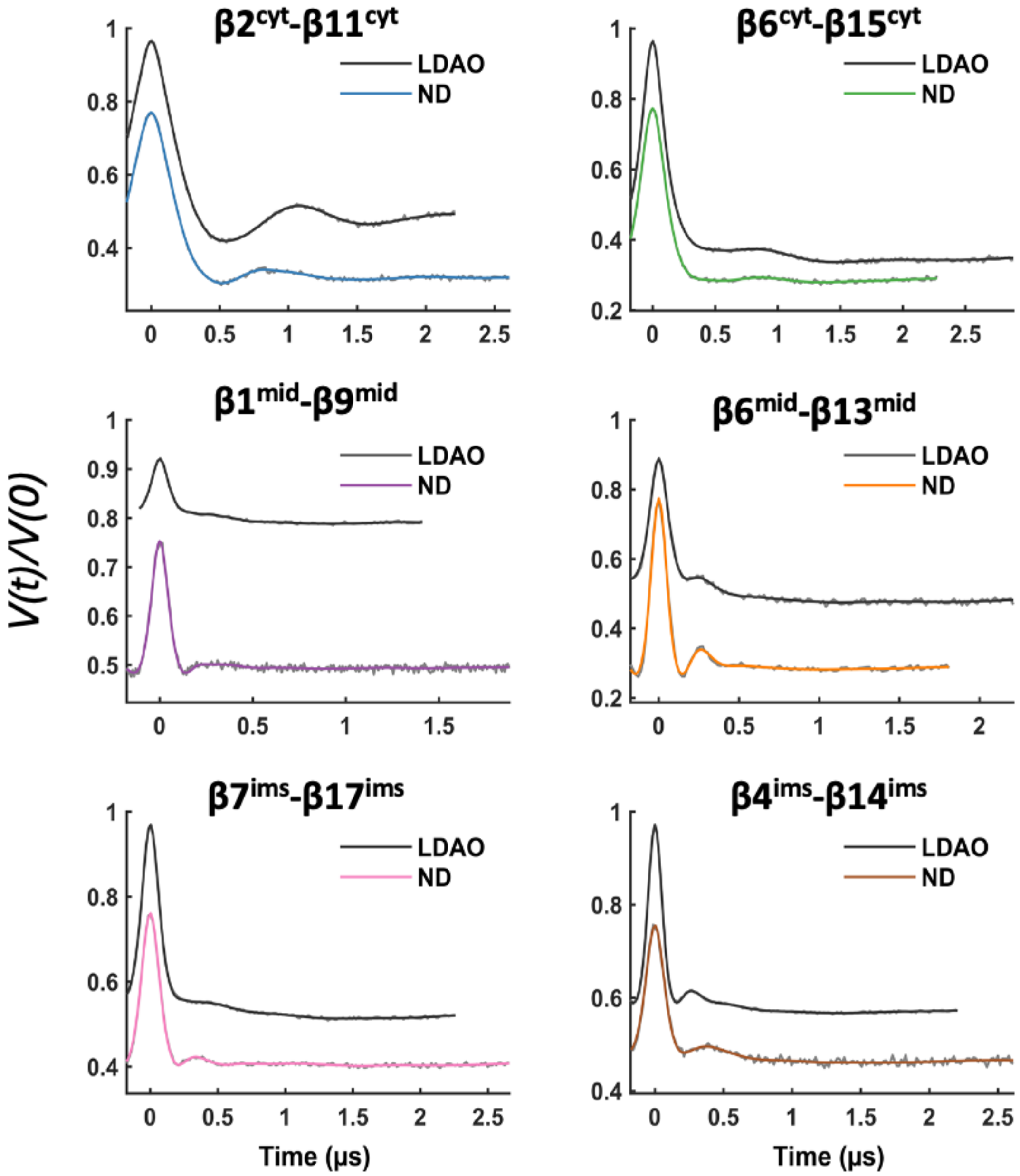

SFig. 2. Four-pulse DEER dipolar evolution traces for the double-cysteine mutants shown in Fig. 2. VDAC samples recorded in LDAO detergent (gray) and lipid nanodiscs (ND; colored traces). Solid lines represent six-Gaussian model–based fits to the experimental dipolar evolution data. Cytosolic (cyt), mid-barrel (mid), and intermembrane space (ims) labeling pairs are indicated in each panel. All traces are background-corrected and normalized as V(t)/V(0). Time is shown in microseconds (μs).

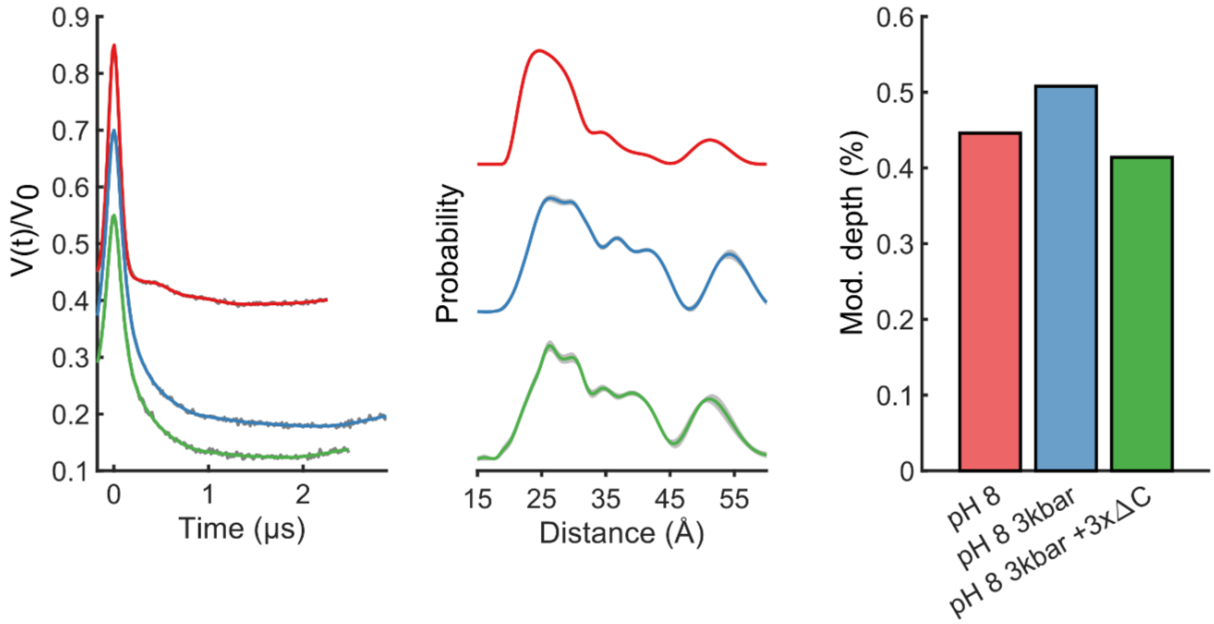

SFig. 3. High pressure enhances intermolecular contributions in detergent-solubilized VDAC. (A) Four-pulse DEER dipolar evolution traces of VDAC1 $\beta7^{ims}$–$\beta17^{ims}$ (119R1–250R1) in LDAO detergent at pH 8 (red), under 3 kbar hydrostatic pressure (blue), and under 3 kbar in the presence of excess unlabeled VDAC to dilute intermolecular spin–spin interactions (green). (B) Corresponding distance distributions obtained from Tikhonov regularization. (C) Modulation depth values under each condition. Application of 3 kbar increases modulation depth and enhances longer-distance components, consistent with pressure-induced oligomerization. Addition of unlabeled VDAC reduces modulation depth under pressure, indicating suppression of intermolecular dipolar contributions.

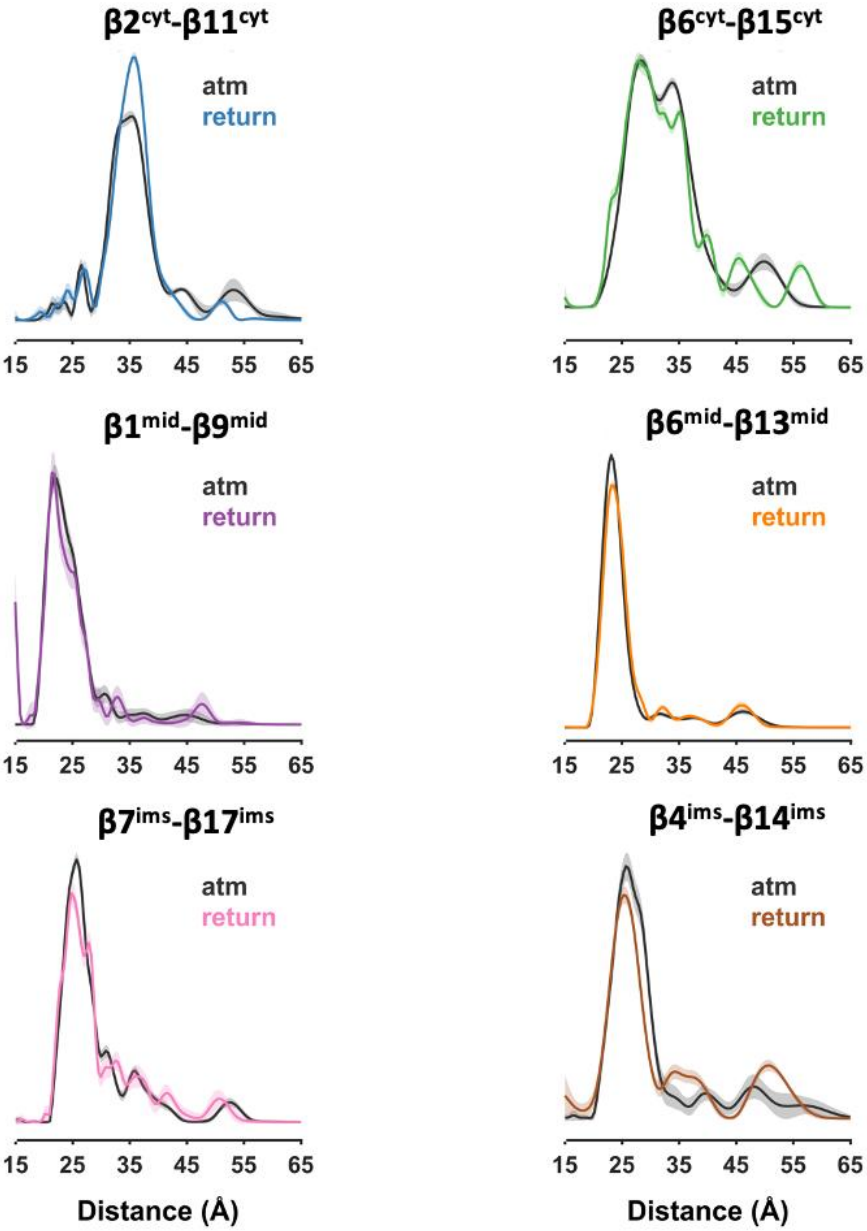

SFig. 4. Reversibility of pressure-induced distance changes.
Distance distributions for the double-cysteine mutants shown in Fig. 3 following decompression to atmospheric pressure (“return”). Colored traces represent the distance distributions after pressure release, overlaid with the corresponding atmospheric-pressure distributions (gray) for comparison. For all labeling positions, the post-pressure profiles closely resemble the initial atmospheric state, indicating that the pressure-induced conformational changes are largely reversible.

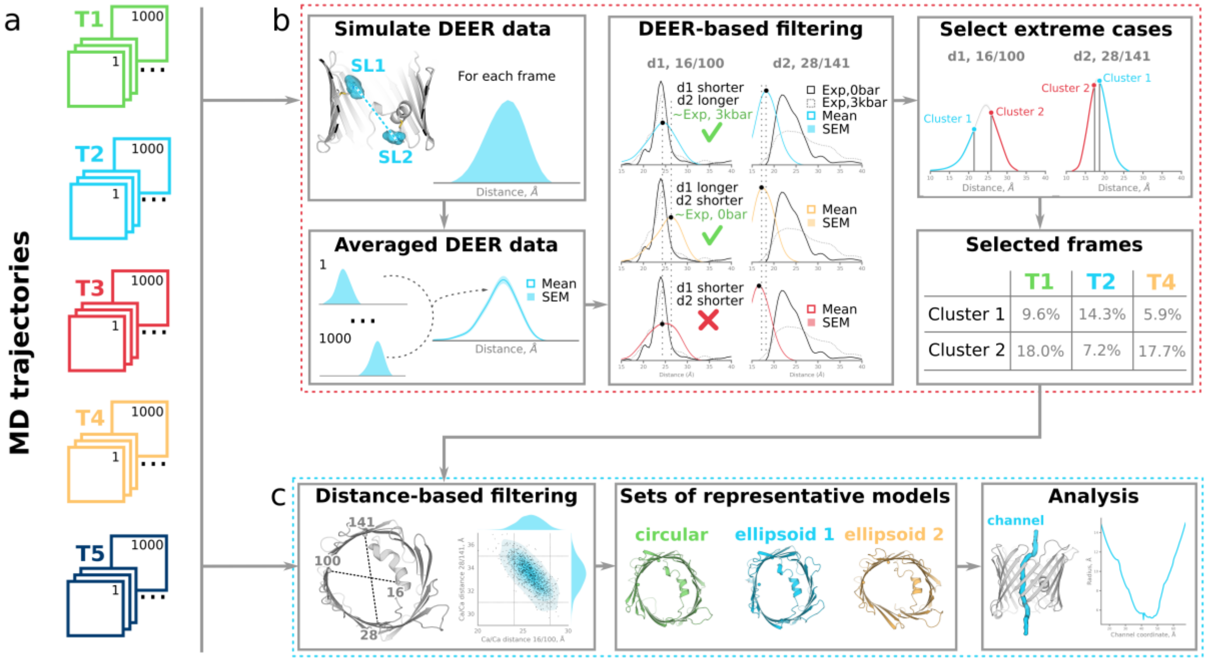

	T1	T2	T4
Cluster 1	9.6%	14.3%	5.9%
Cluster 2	18.0%	7.2%	17.7%

SFig. 5. Workflow for DEER-informed analysis of MD trajectories.
(a) Five independent, unbiased MD trajectories (T1–T5; 1000 frames each) were analyzed to identify conformational states consistent with experimental DEER data. (b) For each trajectory, spin labels were attached in silico and DEER distance distributions were simulated for every frame using chiLife. Simulated DEER traces were averaged across each trajectory and compared with experimental distributions obtained at ambient pressure (0 bar) and under 3 kbar. A DEER-based filtering procedure was applied to select trajectories and frames reproducing the experimentally observed pressure-dependent shifts. Extreme cases corresponding to ambient-like (Cluster 1) and pressure-stabilized (Cluster 2) distance distributions were identified and the corresponding simulation frames were extracted. (c) A subsequent distance-based filtering and clustering step yielded representative structural models corresponding to circular (ambient/open-like) and elliptical (pressure-consistent) conformations. These representative ensembles were used for downstream structural analysis, including pore geometry and channel radius profiling.

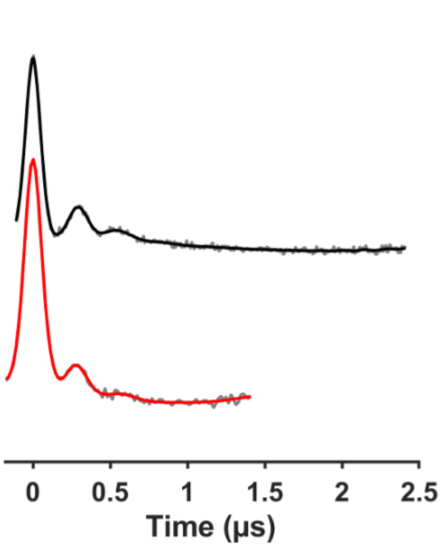

SFig. 6. Acidification does not reproduce pressure-induced conformational changes.
Left: Four-pulse DEER dipolar evolution traces (gray dots) and corresponding six-Gaussian model-based fits (solid lines) for VDAC1 16R1–100R1 in lipid nanodiscs at pH 8 (black) and pH 3.6 (red) under atmospheric pressure. Right: Corresponding distance distributions. No significant shift in the primary distance peak is observed upon acidification, indicating that lowering pH does not mimic the conformational changes induced by hydrostatic pressure.

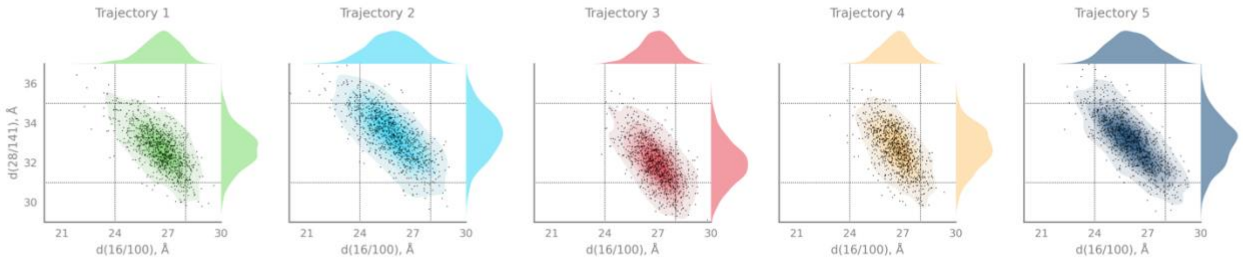

SFig. 7. Averaged simulated DEER distance distributions across independent MD trajectories. Simulated DEER distance distributions for the d(16/100) spin-label pair averaged over all frames within each independent MD trajectory (T1–T5). Shaded regions represent the standard error of the mean (SEM). Corresponding averaged simulated DEER distance distributions for the d(28/141) spin-label pair for each trajectory, with SEM indicated.

Comparison of the trajectory-averaged simulated distributions with experimental DEER data was used to assess which simulations reproduce the pressure-dependent trends—specifically, shorter distances for d(16/100) and longer distances for d(28/141). Based on this analysis, trajectories T1, T2, and T4 were selected for subsequent ensemble characterization.

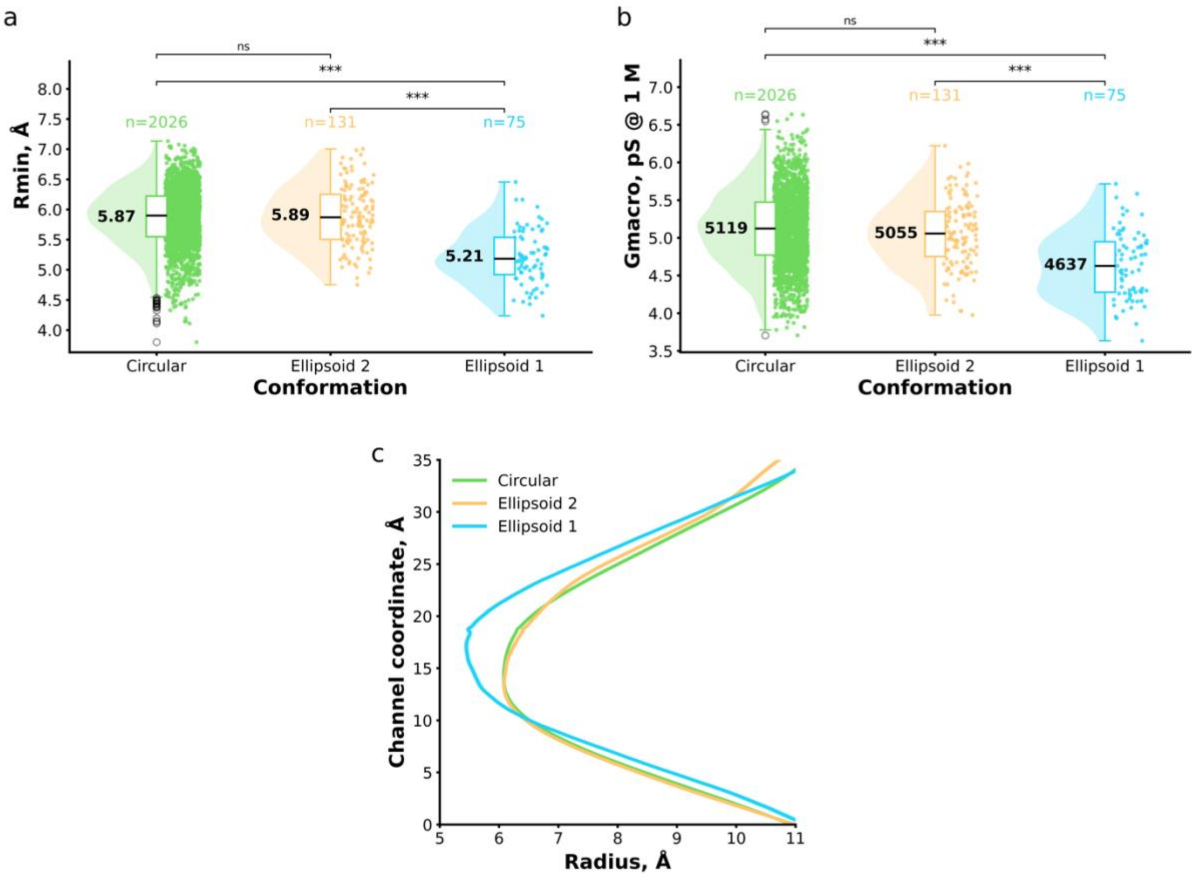

Figure S8. Structural and functional comparison of representative DEER-consistent conformational ensembles.

(a) Distribution of pore bottleneck radii (R_min) calculated using HOLE2 for the circular (green), ellipsoid 2 (orange), and ellipsoid 1 (blue) ensembles derived from the selected MD trajectories (T1, T2, and T4). Individual points represent analyzed frames; box plots indicate median and interquartile range, and violin plots show the underlying distributions. Statistical significance between ensembles is indicated (ns, not significant; ***$p < 0.001$). (b) Estimated single-channel conductance (G_macro) at 1 M salt concentration calculated from pore geometry for the same ensembles. Although differences are observed, conductance values remain substantially higher than those typically associated with electrophysiologically closed states. (c) Axial pore-radius profiles averaged over each ensemble, illustrating a pronounced mid-pore constriction in the ellipsoid 1 conformation relative to the circular and ellipsoid 2 states.

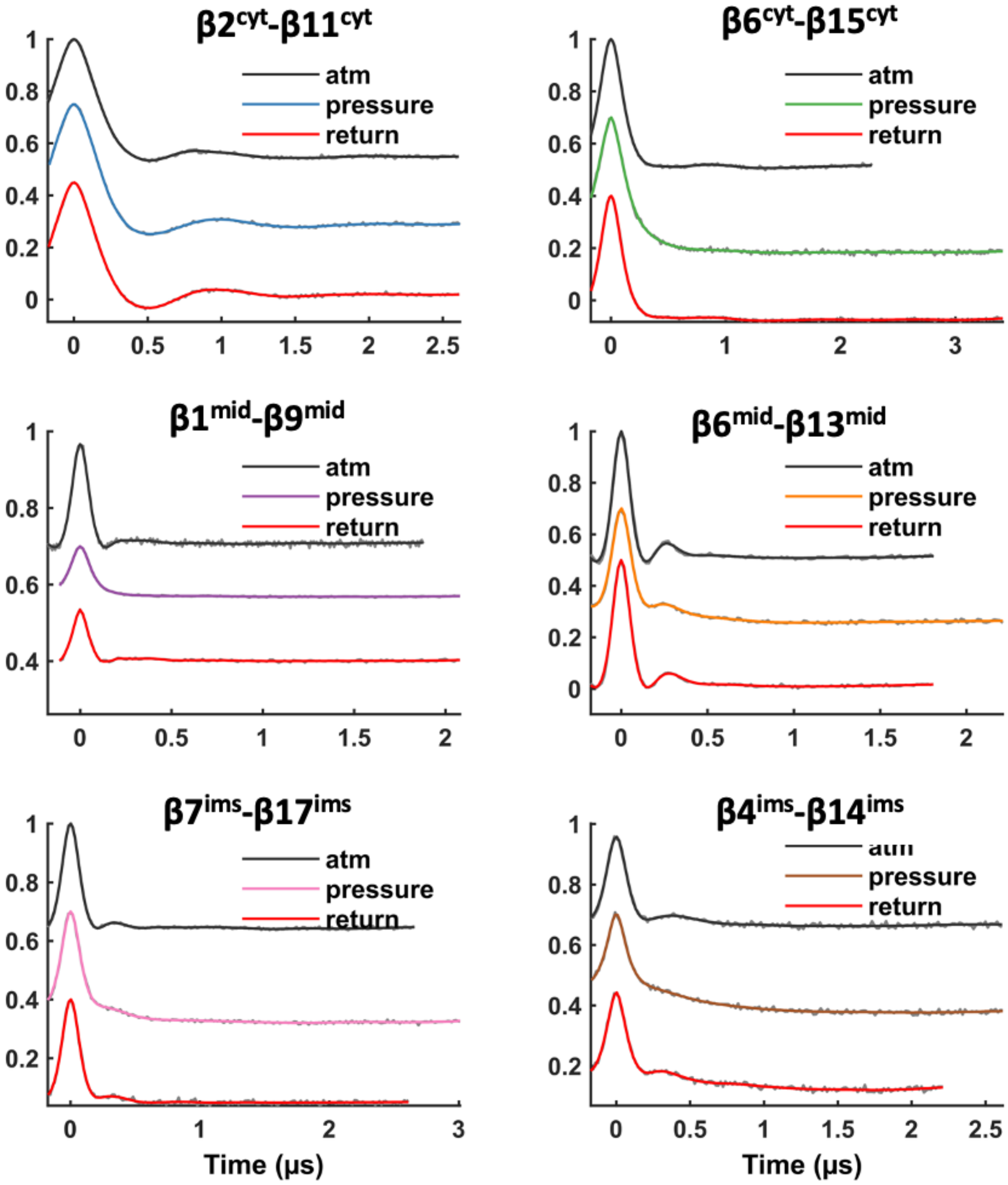

Figure S9: Four-pulse DEER dipolar evolution traces for the double-cysteine mutants shown in Fig. 3. DEER dipolar evolution data (gray dots) and 6-Gaussian model-based fits (plain lines) for the mutants presented Fig. 3.
